# How Fitness Aggregation Methods Affect the Performance of Competitive CoEAs on Bilinear Problems

**DOI:** 10.1007/s00453-025-01313-z

**Published:** 2025-05-15

**Authors:** Mario Alejandro Hevia Fajardo, Per Kristian Lehre

**Affiliations:** University of Birmingham, Birmingham, UK

**Keywords:** Runtime analysis, Competitive co-evolution, Maximin optimisation

## Abstract

Competitive co-evolutionary algorithms (CoEAs) do not rely solely on an external function to assign fitness values to sampled solutions. Instead, they use the aggregation of outcomes from interactions between competing solutions allowing to rank solutions and make selection decisions. This makes CoEAs a useful tool for optimisation problems that have intrinsically interactive domains. Over the past decades, many ways to aggregate the outcomes of interactions have been considered. At the moment, it is unclear which of these is the best choice. Previous research is fragmented and most of the fitness aggregation methods (fitness measures) proposed have only been studied empirically. We argue that a proper understanding of the dynamics of CoEAs and their fitness measures can only be achieved through rigorous analysis of their behaviour. In this work we make a step towards this goal by using runtime analysis to study two commonly used fitness measures. We show a dichotomy in the behaviour of a $(1, \lambda )$ CoEA when optimising a Bilinear problem. The algorithm finds a solution near the Nash equilibrium in polynomial time with high probability if the worst interaction is used as a fitness measure but is inefficient if the average of all interactions is used instead.

## Introduction

In biology the term co-evolution is used to describe a process where two or more species interact with each other reciprocally affecting their success and survival, and consequently their evolution. Co-evolution may happen in a mutualistic relationship, where both species profit from the interaction or a competitive relationship where the species are adversaries (e. g. predator/prey, parasite/host).

Co-evolutionary algorithms (CoEAs) try to mimic biological co-evolution to solve optimisation problems [1]. As in their biological counterparts, they are divided in Cooperative CoEAs [2] and Competitive CoEAs depending on the kind of interactions the solutions take part in. We focus on Competitive CoEAs (we omit the *competitive* label and simply call them CoEAs).

CoEAs have found great success in several applications, ranging from test problems [3, 4] to real-world applications [5–7]. Despite their successes it has been well documented that CoEAs present pathological algorithm dynamics during the optimisation process [1, 8]. These pathological behaviours include, *evolutionary forgetting*: individuals with good characteristics are lost due to lack of selection pressure from the current opponent population, *cyclic dynamics*: traveling through some part of the search space more than once with apparent improvement, *disengagement*: one population overwhelmingly outperforms the other and the fitness gradient disappears, among others. Although these pathologies are well-known, due to the complex population dynamics of CoEAs, they have eluded our understanding. Albeit some remedies have been proposed [5, 6, 9, 10] we argue that without a rigorous understanding of what causes these harmful algorithm dynamics any attempt to solve them has a high likelihood to result futile.

CoEAs use populations of solutions that in each generation interact with each other and later assign a fitness to each solution based on the results (payoffs) of their interactions. A common practice is to aggregate the payoffs into a single numerical fitness value [1], e. g. by averaging the performance outcomes, use the maximum, minimum, median [11]^1^ or more complicated statistics such as fitness sharing [9]. Unfortunately each fitness aggregation method may come with (sometimes harmful) biases depending on the optimisation problem being solved. For example, in cooperative CoEAs averaging interactions was observed to be harmful [12]. Bucci [13] even attributed some of the pathological behaviours encountered in CoEAs to the use of **any** aggregation method.

At the moment, it is unclear when or if certain fitness aggregation methods (also called fitness measures) help the optimisation process or when they result in poor performance. Previous research is fragmented and all of the fitness measures proposed have only been studied empirically.^2^

Due to their complexity, there is little rigorous understanding of the algorithm dynamics in (cooperative and competitive) CoEAs, although recent years have seen an increase in the number of studies addressing these challenges through theoretical means. Jansen and Wiegand [14] rigorously analysed the runtime of a cooperative CoEA on *separable* functions and showed that problem separability does not guarantee a speedup over traditional EAs. Lehre and Lin [15] showed that the cooperative CoEA CC-$(1+1)$ EA solves all linear functions in the same asymptotic time as the $(1+1)$ EA.

For competitive CoEAs, Lehre [16] analysed for the first time the runtime of a competitive CoEA, the PDCoEA on some instances of the pseudo-Boolean Bilinear problem, showing that given the correct parameters the algorithm finds an $\varepsilon$-approximation efficiently, but an incorrect parameter setting leads to exponential runtime. Fajardo et al. [17] analysed the runtime and total regret of a CoEA named RLS-PD on some instances of the pseudo-Boolean Bilinear problem. The authors showed that, despite finding the optimum in $O(n^{1.5})$, the algorithm quickly forgets this solution and stay far away from it, resulting in a large regret due to the absence of populations. In a follow up study, Lehre and Lin [18] provided a novel drift theorem that gives precise exponential tail-bounds on the runtime of algorithms given positive, weak, zero and even negative drift. With this new theorem, the authors showed that the runtime of RLS-PD and the RWAB bandit algorithm on Bilinear is highly concentrated. Benford and Lehre [19] analysed CoEAs within a class of symmetric zero-sum games, proving that populations are necessary for efficient runtimes, and showed that a coevolutionary UMDA can find the optimum in $O(n(\log n)^2)$. Lehre and Lin [20] investigated the difference between EAs and CoEAs on binary test-based problems. The authors showed that for the pseudo-Boolean problem Diagonal a $(1,\lambda )$ CoEA with appropriate parameter values is able to find an $\varepsilon$ approximation efficiently while a $(1,\lambda )$ EA needs superpolynomial time. Fajardo and Lehre [21] studied how the correct set of solutions used for evaluation can affect the runtime of CoEAs, showing that a good diversity of rankings is beneficial on the pseudo-Boolean Bilinear problem. Based on this the authors proposed and analysed two new CoEAs that maintain a high diversity of rankings. Both algorithms optimise Bilinear in polynomial time.

This work is a step forward towards a rigorous understanding of how different fitness measures can alleviate or aggravate the pathological behaviours of CoEAs. We consider the ${(1 , \lambda )}~\textrm{CoEA}$ (Algorithm 1) on a class of Bilinear problems with an infinite discrete search space (cf. Sect. 2.2). Bilinear is an important class of Maximin-problems because it is thought that the structure of the Bilinear problem class is similar to the underlying structure of many applications such as the training of GANs [22]. Additionally, it is a challenging class of problems because they have intransitive properties and the continuous versions are known to result in cyclic behaviour for some gradient-based algorithms [23–25]. Here, we ask whether the gradient-free ${(1 , \lambda )}~\textrm{CoEA}$ is able to find a suitable solution despite the intransitivity of the problem and what is the role of the fitness measures in the performance of the algorithm.

In Sects. 3 and 4 we characterise how the fitness measures assign fitness values based on the current population and how the mutation operator creates solutions as a stepping stone of the following theoretical analysis.

Afterwards, in Sect. 5 we show that the ${(1 , \lambda )}~\textrm{CoEA}$ using the average of interactions as fitness measure not only results in a cyclic behaviour, but every cycle the algorithm moves away from the optimum in expectation. This result in an infinite expected time to find an optimal solution.

In sharp contrast, in Sect. 6 we show that the ${(1 , \lambda )}~\textrm{CoEA}$ using the worst interaction as fitness measure is efficient, finding a solution near the optimum in polynomial time. We note that the algorithm still presents a cycling behaviour but the fitness measure helps alleviate the problem.

Finally, we present an experimental analysis where we test the applicability of our theoretical analysis to various search domains. Our observations demonstrate that our theoretical analysis successfully translates into these different search domains. That is, for all search domains studied, using the average as a fitness measure results in large runtimes and using the minimum as a fitness measure results in efficient runtimes.

A preliminary conference version of our results appeared in [26]. The findings presented in this manuscript have undergone substantial improvements since then. In [26] we showed that w. o. p. the number of evaluations needed by the ${(1 , \lambda )}~\textrm{CoEA}$ using the average of interactions as fitness measure was exponential. This was estimated using the negative drift theorem with scaling ([27, Theorem 2]) but we found a mistake in the application of this theorem. In this work we have identified an alternative approach and showed that the runtime is infinite in expectation.

Another substantial change is that in our previous results, when analysing the fitness measure that uses the worst interaction as fitness, we showed that a modified version of the ${(1 , \lambda )}~\textrm{CoEA}$ that creates offspring deterministically around the parents, finds the optimum in polynomial time. In this manuscript we analyse the ${(1 , \lambda )}~\textrm{CoEA}$ that creates offspring at random instead. However, a drawback of this analysis is that we can only guarantee the algorithm finds solutions near the optimum efficiently if the offspring population size is proportional to the initial distance.

## Preliminaries

We study how different fitness aggregation methods (fitness measures) affect the performance of CoEAs on Bilinear problems. In particular we study the expected runtime of the ${(1 , \lambda )}~\textrm{CoEA}$ (Algorithm 1) with two different fitness measures on the class of Bilinear problems.

The ${(1 , \lambda )}~\textrm{CoEA}$ as defined in Algorithm 1 optimises a Maximin-optimisation problem with any domain $\mathcal {X}, \mathcal {Y}$ (*strategy space* or *search space*) and use any mutation operators $\text {mut}_x(\cdot ):\mathcal {X} \rightarrow \mathcal {X}$ and $\text {mut}_y(\cdot ):\mathcal {Y} \rightarrow \mathcal {Y}$. In concordance to the algorithm, the class of Bilinear problems can be defined in different search spaces too. We explore further this problem class in Sect. 2.2. For our theoretical analysis we consider $\mathcal {X} =\mathcal {Y} = \mathbb {Z}$ and the mutation operator used is discussed in detail in Sect. 4.

We choose the search spaces $\mathcal {X} = \mathcal {Y} = \mathbb {Z}$ for several reasons. The domain is discrete, therefore, gradient-based algorithms do not work denoting the importance of gradient-free algorithms such as CoEAs. Despite being a discrete domain, $\mathbb {Z}$ is infinite, which allows the creation of *real* unbiased mutation operators, in the sense that movement towards one direction of the search space is not limited by the search space itself making it less likely to happen. This reason is particularly important in this study, because we want to focus our study on how the fitness measures affect the performance of CoEAs and this allows us to decouple the behaviour of the selection mechanism (based on the fitness measures) from the inherent tendency away from the boundaries of the search space that a mutation operator in a finite search space have.

### The $(1, \lambda )$ CoEA

The ${(1 , \lambda )}~\textrm{CoEA}$ uses a parent population of size one for each search space $\mathcal {X}, \mathcal {Y}$. In each generation the algorithm creates $2 \lambda$ offspring by mutating each parent $\lambda$ times. Later it uses a fitness measure to assign a fitness value to every offspring in one population depending on their interactions with the other offspring population.

**Algorithm 1:**
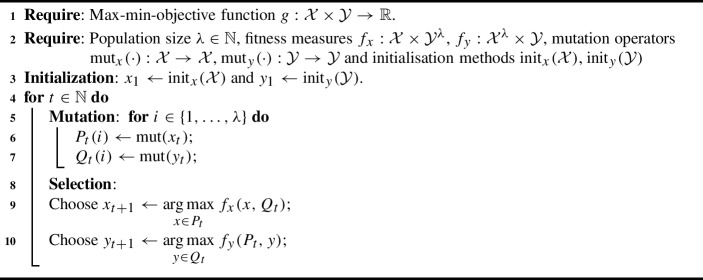
$$(1, \lambda )$$ CoEA

We consider two fitness measures *f*:

- Individual vs all (average) $f^{\textrm{avg}}$: Each individual in a population is evaluated against every other individual in its competing population, and the average^3^ of the evaluations is used to determine the individuals’ fitness. That is,
  $$\begin{aligned} f^{\textrm{avg}}_{\mathcal {X}}(x,Q_t)=\frac{1}{\lambda }\sum _{j=1}^{\lambda } g(x,Q_t(j)),\\ f^{\textrm{avg}}_{\mathcal {Y}}(P_t,y)=-\frac{1}{\lambda }\sum _{j=1}^{\lambda } g(P_t(j),y). \end{aligned}$$
- Individual vs all (worst) $f^{\textrm{wrs}}$: Each individual in a population is evaluated against every other individual in its competing population, and the worst of the evaluations is used to determine the individuals’ fitness. That is,
  $$\begin{aligned} f^{\textrm{wrs}}_{\mathcal {X}}(x,Q_t)=\min _{j\in [\lambda ]} g(x,Q_t(j)),\\ f^{\textrm{wrs}}_{\mathcal {Y}}(P_t,y)=-\max _{j\in [\lambda ]} g(P_t(j),y). \end{aligned}$$

### Bilinear

The class of Bilinear problems are a simple and well-defined class of Maximin-optimisation problems. The Bilinear problems have been extensively used to understand the behaviour of Maximin-optimisation algorithms (e.g. [23–25]) and it was recently used by Lehre [16] to analyse a population-based CoEA called PDCoEA. Vlatakis-Gkaragkounis et al. [22] suggest that the structure of these problems imitates the underlying structure of important applications such as training GANs.

The general form of Bilinear considers an *n*-dimensional (continuous or discrete) domain for the solutions *x*, *y*. Since we consider the 1-dimensional search spaces $\mathcal {X} =\mathcal {Y} = \mathbb {Z}$, we give a simpler form.

$$\begin{aligned} \textsc {Bilinear} _{\alpha ,\beta }(x, y):= x y -\alpha x - \beta y. \end{aligned}$$

**Fig. 1:**
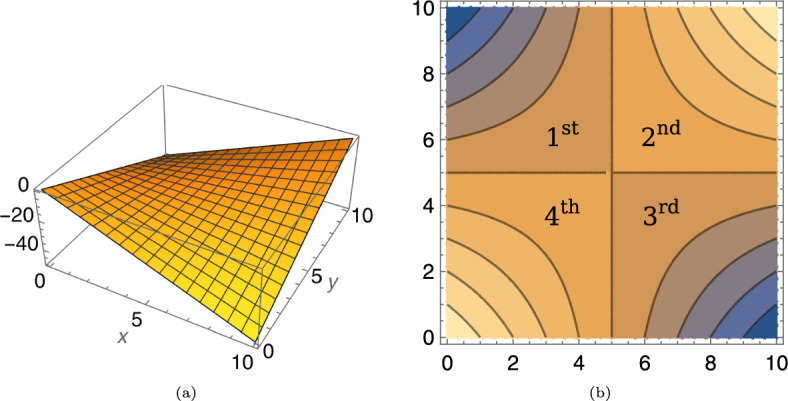
Bilinear for $\alpha =5$ and $\beta =5$ **a** 3D-graph **b** Contour with the quadrants enumerated

The parameters $\alpha$ and $\beta$ denote where the Maximin-solutions (also called Nash equilibria or optimal solutions) are found. Figure 1 shows the 3D-graph of the function and its contour plot.

During our analysis, we divide the search space into four *quadrants* (Fig. 1b). We say that a pair of search points (*x*, *y*) is in:

- The first quadrant if $x<\beta \wedge \alpha \le y$,
- The second quadrant if $\beta \le x \wedge \alpha <y$,
- The third quadrant if $\beta <x \wedge y \le \alpha$, and
- The fourth quadrant if $x \le \beta \wedge y <\alpha$.

### Notation and Probability Tools

For any natural number $n\in \mathbb {N}$ we define $[n]:=\{1,2,\dots ,n\}$. Let $W_1, \dots , W_n$ be i. i. d. random variables. Then we denote the maximum (*n*-th order statistic) of these variables as $W_{(n)}$. We write $\textrm{E}_t\mathord {\left[ X\right] }$ to denote the expectation $\textrm{E}\mathord {\left[ X\mid \mathcal {F}_t\right] }$ of a random variable *X* conditional on the filtration $\mathcal {F}_t$.

By $\mathcal {G}(p)$ we denote the geometric distribution with parameter $p\in [0,1]$. The geometric distribution is often defined in two different ways, we define it as the probability distribution of the number of failures before the first success of Bernoulli trials with success probability *p*. Then, for $X\sim \mathcal {G}(p)$ we have

$$\begin{aligned} \textrm{Pr}\left[ X=k\right] =(1-p)^k p, \quad \textrm{E}\mathord {\left[ X\right] }=\frac{1-p}{p}. \end{aligned}$$

For a pair of solutions $x_t$ and $y_t$ at time *t* we define the distance to the optimum as the Manhattan distance $M(x_t, y_t):=\left|x_t-\beta \right|+\left|y_t-\alpha \right|$ and for convenience we refer to it as $M_t$.

In Definition 2.1 we introduce notation for characteristics of the populations at time *t*.

#### Definition 2.1

For populations $P_t\in \mathcal {X}$ and $Q_t\in \mathcal {Y}$ we define:

$$\begin{aligned} X_t&:=\displaystyle \sum _{x\in P_t}\left( x-\beta \right)&\overline{X}_t&:=\displaystyle \sum _{x\in P_t}\left|x-\beta \right|\\ Y_t&:=\displaystyle \sum _{y\in Q_t}\left( y-\alpha \right)&\overline{Y}_t&:=\displaystyle \sum _{y\in Q_t}\left|y-\alpha \right|\\ x_{\min }&:=\min _{x\in P_t}{x}&x_{\max }&:=\max _{x\in P_t}{x} \\ y_{\min }&:=\min _{y\in Q_t}{y}&y_{\max }&:=\max _{y\in Q_t}{y} \\ P^-_t&:=\{x\in P_t \mid x\le \beta \}&P^+_t&:=\{x\in P_t \mid x\ge \beta \}\\ Q^-_t&:=\{y\in Q_t \mid y\le \alpha \}&Q^+_t&:=\{y\in Q_t \mid y\ge \alpha \}\\ x^+_{\min }&:=\min _{x\in P_t^+}{x}&x^-_{\max }&:=\max _{x\in P_t^+}{x} \\ y^+_{\min }&:=\min _{y\in Q_t^+}{y}&y^-_{\max }&:=\max _{y\in Q_t^+}{y}\\ x^*_{\min }&:=\underset{x\in P_t}{\arg \min }{\, \left|x-\beta \right| }&y^*_{\min }&:=\underset{y\in Q_t}{\arg \min }{\, \left|y-\alpha \right| } \end{aligned}$$

### Drift Theorems

Drift analysis is one of the most useful tools to analyse evolutionary algorithms [28]. Many of these theorems are also useful for coevolutionary algorithms, and we employ them in our analyses. For convenience, we state the upper and lower additive drift theorems used here.

#### Theorem 2.2

(Upper additive drift, unbounded [29]) Let $\alpha \le 0$, let $(X_t)_{t\in \mathbb {N}}$ be random variables over $\mathbb {R}$, and let $T=\inf \{t\mid X_t\le 0\}$. Furthermore, suppose that,

- For all $t\le T$, it holds that $X_t\ge \alpha$, and that
- There is some value $\delta >0$ such that, for all $t<T$, it holds that $X_t-\textrm{E}\mathord {\left[ X_{t+1}\mid X_0\dots X_t\right] }\ge \delta$.

Then

$$\begin{aligned} \textrm{E}\mathord {\left[ T\mid X_0\right] } \le \frac{X_0-\alpha }{\delta }. \end{aligned}$$

#### Theorem 2.3

(Lower additive drift, expected bounded step size [29]) Let $(X_t)_{t\in \mathbb {N}}$ be random variables over $\mathbb {R}$, and let $T=\inf \{t\mid X_t\le 0\}$. Furthermore, suppose that,

- There is some value $\delta >0$ such that, for all $t<T$, it holds that $X_t-\textrm{E}\mathord {\left[ X_{t+1}\mid X_0\dots X_t\right] }\le \delta$, and that
- There is some value $c>0$ such that, for all $t<T$, it holds that $\textrm{E}\mathord {\left[ \left|X_{t+1}-X_{t}\right|\mid X_0\dots X_t\right] }\le c$.

Then

$$\begin{aligned} \textrm{E}\mathord {\left[ T\mid X_0\right] } \ge \frac{X_0-\textrm{E}\mathord {\left[ X_T\mid X_0\right] }}{\delta } \ge \frac{X_0}{\delta }. \end{aligned}$$

## Analysis of the Fitness Measures on Bilinear

In this section we focus our attention on how the fitness measures assign fitness to individual solutions. In particular we explore what solutions have the *best* fitness with respect to each fitness measure on Bilinear. We start with the fitness measure $f^{\textrm{avg}}$.

### Lemma 3.1

(Fitness Based on Averaging Interactions) Consider two populations of solutions $P_t$ and $Q_t$ and the fitness measure $f^{\textrm{avg}}$ on Bilinear. Then $f^{\textrm{avg}}$ assigns the highest fitness to all solutions in the sets $\xi \subseteq P_t, \Upsilon \subseteq Q_t$ defined as,

$$\begin{aligned} \xi = {\left\{ \begin{array}{ll} \{x\mid x=x_{\min }\} &  \textrm{if}\ Y_t<0,\\ \{x\mid x=x_{\max }\} &  \textrm{if}\ Y_t>0,\\ P_t &  \textrm{otherwise}, \end{array}\right. } \end{aligned}$$

and

$$\begin{aligned} \Upsilon = {\left\{ \begin{array}{ll} \{y\mid y=y_{\max }\} &  \textrm{if}\ X_t<0,\\ \{y\mid y=y_{\min }\} &  \textrm{if}\ X_t>0,\\ Q_t &  \textrm{otherwise}. \end{array}\right. } \end{aligned}$$

### Proof

We use the fitness measure $f^{\textrm{avg}}$ to assign a value to each individual. For every individual $x\in P_t$ its fitness measure is

$$\begin{aligned} f(x,Q_t)&= \frac{1}{\lambda }\sum _{y\in Q_t} \left( x y-\alpha x - \beta y\right) = \frac{1}{\lambda } \left( \sum _{y\in Q_t} x (y-\alpha )- \sum _{y\in Q_t} \beta y\right) . \end{aligned}$$

In the same manner, for every individual $y\in Q_t$ its fitness measure is

$$\begin{aligned} f(y,P_t)&= -\frac{1}{\lambda } \sum _{x\in P_t} \left( x y-\alpha x - \beta y \right) = - \frac{1}{\lambda } \left( \sum _{x\in P_t}y (x - \beta ) - \sum _{x\in P_t} \alpha x\right) . \end{aligned}$$

Since we are only comparing their fitness, if we add, subtract or multiply a positive number to all the fitness values the comparison stays equal. Therefore, we can change the fitness measures for

$$\begin{aligned} f(x,Q_t)&= x \sum _{y\in Q_t} (y-\alpha ) = xY_t \quad \text {and}\\ f(y,P_t)&= -y \sum _{x\in P_t} (x - \beta ) = -yX_t, \end{aligned}$$

without changing the results of this comparisons. With this result we can see that if $Y_t>0$ the individual *x* with the largest value has the highest fitness. Similarly, if $Y_t<0$ the individual with the smallest value has the highest fitness. And, if $Y_t=0$ all individuals *x* have the same fitness. Using the same arguments and noting that the sign in $f(y,P_t)$ reverse the order of fitness yields the statement for *y*. $\square$

The most important thing to note from Lemma 3.1 is that the decisions are made depending on the distance of all solutions to their respective optimum and that most of the time the solutions with the highest fitness are the solutions farthest to their parent.

Lemma 3.2 describes the fitness measure $f^{\textrm{wrs}}$. The behaviour of this fitness measure is noticeably more complex than $f^{\textrm{avg}}$.

### Lemma 3.2

(Fitness Based on Worst Interactions) Consider two populations of solutions $P_t$ and $Q_t$ and the fitness measure $f^{\textrm{wrs}}$ on Bilinear. Then $f^{\textrm{wrs}}$ assigns the highest fitness to all solutions in the sets $\xi \subseteq P_t, \Upsilon \subseteq Q_t$ defined as,
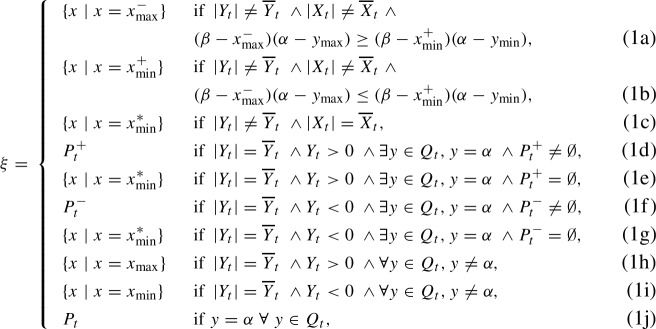
 and 
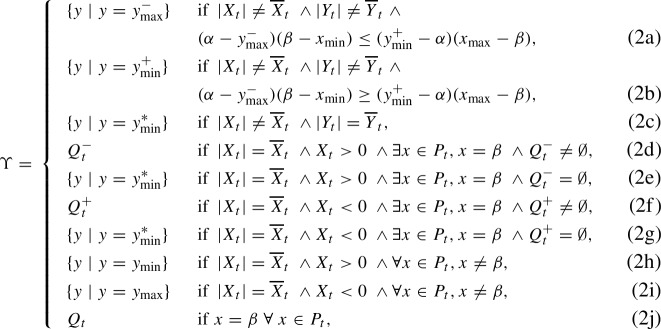
 respectively.

### Proof

In this proof we show what is the individual *x* with highest fitness and note that the same arguments (with slight variations) result in the individual *y* with highest fitness. Given that there are several cases we go through each of them in the order that they appear in the statement of Lemma 3.2.

For simplicity, let $x_i:=P_t(i)$ and $y_i:=Q_t(i)$, then

$$\begin{aligned} f^{\textrm{wrs}}_{\mathcal {X}}(x_i,Q_t)=\min _{i\in [\lambda ]}\{g(x_i,y_i)\}=\min _{i\in [\lambda ]}\{x_iy_i-\alpha x_i-\beta y_i\}. \end{aligned}$$

We note that the condition $\left|X_t\right|\ne \overline{X}_t$ implies that there are individuals in population $P_t$ with both $x_i<\beta$ and $x_i>\beta$ and $\left|X_t\right| = \overline{X}_t$ implies that all individuals in population $P_t$ have either $x_i\le \beta$ or $x_i\ge \beta$. The same relations apply for the population $Q_t$.

Due to the note above the statement $\left|X_t\right| \ne \overline{X}_t\;\wedge \left|Y_t\right|\ne \overline{Y}_t$ in Conditions (1a) and (1b) imply that the pairs of individuals in the Cartesian product $P_t\times Q_t$ lie in the four quadrants of the search space. For any $x_i<\beta$ the minimum value is attained for the *y* with the highest value, for any $x_i>\beta$ the minimum value is attained for the *y* with the lowest value. Then, if we only consider $x_i\le \beta$ the fitness of every individual is

$$\begin{aligned} f^{\textrm{wrs}}_{\mathcal {X}}(x_i,Q_t) = x_iy_{\max }-\alpha x_i-\beta y_{\max }. \end{aligned}$$

and among them, the individual with highest fitness is the individual nearest to $\beta$ with $x_i\le \beta$, that is $x_{\max }^-$. Therefore its fitness is

3 $$\begin{aligned} f^{\textrm{wrs}}_{\mathcal {X}}(x_{\max }^-,Q_t)= x_{\max }^-y_{\max }-\alpha x_{\max }^- -\beta y_{\max }. \end{aligned}$$

If we only consider $x_i\ge \beta$ the fitness of every individual is

4 $$\begin{aligned} f^{\textrm{wrs}}_{\mathcal {X}}(x_i,Q_t)= x_iy_{\min }-\alpha x_i-\beta y_{\min }. \end{aligned}$$

and among them, the individual with highest fitness is the individual nearest to $\beta$ with $x_i\ge \beta$, that is $x_{\min }^+$. Therefore its fitness is

$$\begin{aligned} f^{\textrm{wrs}}_{\mathcal {X}}(x_{\min }^+,Q_t)= x_{\min }^+y_{\min }-\alpha x_{\min }^+ -\beta y_{\min }. \end{aligned}$$

Conditions (1a) and (1b) also rely on the following values:

$$\begin{aligned} (\beta -x_{\max }^-)(\alpha -y_{\max })&=\alpha \beta +x_{\max }^-y_{\max } -\alpha x_{\max }^--\beta y_{\max }\\ (\beta -x_{\min }^+)(\alpha -y_{\min })&=\alpha \beta +x_{\min }^+y_{\min } -\alpha x_{\min }^+-\beta y_{\min }. \end{aligned}$$

Then,

$$\begin{aligned} (\beta -x_{\max }^-)(\alpha -y_{\max })&\ge (\beta -x_{\min }^+)(\alpha -y_{\min })\\ \Leftrightarrow \qquad f^{\textrm{wrs}}_{\mathcal {X}}(x_{\max }^-,Q_t)&\ge f^{\textrm{wrs}}_{\mathcal {X}}(x_{\min }^+,Q_t), \end{aligned}$$

and $x_{\max }^-$ has the highest fitness in the population. If the inequality is in the other direction $x_{\min }^+$ has the highest fitness. This proves the first two cases.

The Condition (1c) imply that the pairs of individuals in the Cartesian product $P_t\times Q_t$ lie in the two left or right quadrants of the search space. Due to Eqs. (3) and (4) the highest is individual is the closest to $\beta$ showing the third case.

The Conditions (1d) and (1e) imply that the pairs of individuals in the Cartesian product $P_t\times Q_t$ lie in the top part of the search space and there is an optimal individual in $Q_t$. Additionally if $P_t^+= \emptyset$ the pairs of individuals only lie in the first quadrant ($x_i<\beta$) then the minimum payoff of all individuals is attained with $y_{\max }$ and as in Eq. (3) the highest fitness is attained for $x_{\max }^-$. If $P_t^+\ne \emptyset$ then there are some pairs of individuals in the second quadrant ($x_i\ge \beta$). In this case the fitness of individuals in the second quadrant is higher than the ones in the first quadrant and their minimum payoff is attained for $y=\alpha$ giving them all the same fitness,

$$\begin{aligned} f^{\textrm{wrs}}_{\mathcal {X}}(x_i,Q_t)=\min _{y\in Q_t}\{x_iy_i-\alpha x_i-\beta y_i\}=x_i \alpha -\alpha x_i-\beta \alpha = -\beta \alpha . \end{aligned}$$

This proves the fourth and fifth case. For the sixth and seventh case the arguments are the same but mirrored for the bottom part of the search space.

Conditions (1 h) and (1i) imply that the pairs of individuals in the Cartesian product $P_t\times Q_t$ lie in exactly one quadrant and there are no optimal individuals in $Q_t$. The Condition (1 h) refers to the first and second quadrant and Condition (1i) refers to the third and fourth quadrant.

For Condition (1 h) since $y_i>\alpha$ the fitness of all individuals is

$$\begin{aligned} f^{\textrm{wrs}}_{\mathcal {X}}(x_i,Q_t)&=\min _{y\in Q_t}\{x_iy_i-\alpha x_i-\beta y_i\}\\&=x_i y_{\max } - \alpha x_i-\beta y_{\max }\\&=x_i(y_{\max } - \alpha )-\beta y_{\max }, \end{aligned}$$

therefore, the individual *x* with highest value has the highest fitness. For Condition (1i) $y_i<\alpha$ and similarly the fitness of all individuals is

$$\begin{aligned} f^{\textrm{wrs}}_{\mathcal {X}}(x_i,Q_t)&=\min _{y\in Q_t}\{x_iy_i-\alpha x_i-\beta y_i\}\\&=x_i y_{\min } - \alpha x_i-\beta y_{\min }\\&=x_i(y_{\min } - \alpha )-\beta y_{\min }. \end{aligned}$$

Since $(y_{\min } - \alpha )<0$, the individual *x* with lowest value has the highest fitness.

Finally, the last case says that all $y\in Q_t$ have $y=\alpha$, therefore all individuals in $P_t$ have the same fitness

$$\begin{aligned} f^{\textrm{wrs}}_{\mathcal {X}}(x_i,Q_t)&=\min _{y\in Q_t}\{x_iy_i-\alpha x_i-\beta y_i\}=x_i \alpha - \alpha x_i-\beta \alpha =-\beta \alpha .&\end{aligned}$$

**Fig. 2:**
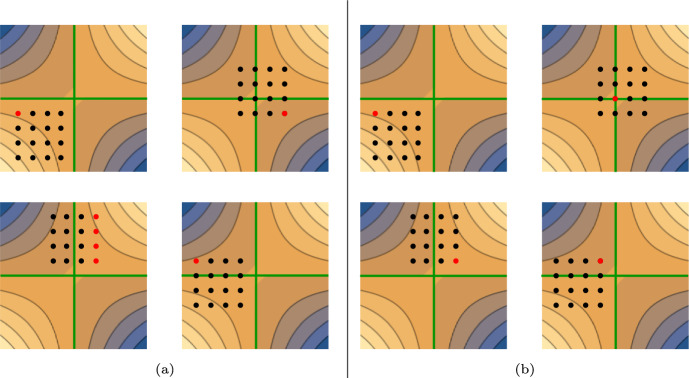
Example populations $P\times Q$ with the pair of individuals with highest fitness value highlighted (red point) for fitness measures $f^{\textrm{avg}}$ **a** and $f^{\textrm{wrs}}$ **b**. $x=\beta$ and $y=\alpha$ are denoted by the green lines and the optimum is at the crossing of these lines

Figure 2 shows some example populations and their highest individuals for fitness measures $f^{\textrm{avg}}$ (a) and $f^{\textrm{wrs}}$ (b). The most interesting example is when $P\times Q$ comprises the four quadrants and the optimal solution is included (top right in Fig. 2a and b). In this case $f^{\textrm{wrs}}$ correctly assigns the highest fitness to the optimum but $f^{\textrm{avg}}$ does not. When $P\times Q$ comprises two quadrants (bottom left and right in Fig. 2a and b) $f^{\textrm{avg}}$ tend to assign the highest fitness to individuals farther away from the optimum than the ones chosen by $f^{\textrm{wrs}}$. Finally if $P\times Q$ comprises one quadrant (top left in Fig. 2a and b) both fitness measures behave identically: the individual with highest fitness is always the corner *ahead* in a clockwise direction.

## Analysis of the Mutation Operator

The ${(1 , \lambda )}~\textrm{CoEA}$ analysed here uses the following simple mutation operator, which is called geometric mutation. For a parent *x* it creates the *i*-th offspring $x'$ by sampling $W_i\sim \mathcal {G}(p)$ and adding or subtracting that value to the parent. To simplify the analysis the mutation operator creates exactly $\lambda /2$ offspring as $x'=x+W_i$ and the other $\lambda /2$ offspring as $x'=x-W_i$. This could be randomised maintaining the same expected number of offspring increasing and decreasing value from the parent as follows: with probability 1/2 the operator adds $W_i$ and subtracts $W_i$ otherwise. Although we believe our analysis hold for the randomised case, we decided not to add it to avoid over-complicating the already complex analysis.

An important thing that we learnt in Sect. 3 is that for both fitness measures the offspring with highest fitness is often the farthest away from the parent (with respect to one of the directions). Therefore, we need to characterise the behaviour of the maximum value of several geometric random variables. In the following lemma we study its expected value.

### Lemma 4.1

Let $0<p<1$ be a constant, $\psi \in \mathbb {N}$, $W_i\sim \mathcal {G}(p)$ with $1 \le i\le \psi$ and $W_{(\psi )}:=\displaystyle \max _{i\in [\psi ]}\{W_i\}$ then

5 $$\begin{aligned} \left( 1/2-e^{-1}\right) \lfloor \log _{\frac{1}{1-p}} \psi \rfloor \le \textrm{E}\mathord {\left[ W_{(\psi )}\right] }&\le \Big \lceil \log _{\frac{1}{1-p}} \psi \Big \rceil +\frac{1-p}{p}, \end{aligned}$$

6 $$\begin{aligned} {\textrm{E}\mathord {\left[ W^{(\psi )}\mid W^{(\psi )}\ge j\right] } }&\le j+\textrm{E}\mathord {\left[ W_{(\psi (1-p)^{j}+1)}\right] }. \end{aligned}$$

The lower bound in (5) also holds when conditioning on all $W_i\le 4 \log _{\frac{1}{1-p}} \psi$ if $\psi >1$.

### Proof

We start with the unconditional expectation first, and deal with the conditional expectation later. For the lower bound we compute the expectation as

$$\begin{aligned} \textrm{E}\mathord {\left[ W_{(\psi )}\right] } {= \sum _{k=1}^{\infty }k \cdot \textrm{Pr}\left[ W_{(\psi )}=k\right] =\sum _{k=1}^{\infty }k \cdot \left( \textrm{Pr}\left[ W_{(\psi )}\ge k\right] -\textrm{Pr}\left[ W_{(\psi )}\ge k+1\right] \right) } \end{aligned}$$

Since $W_{(\psi )}$ is the maximum of $\psi$ random variables, $\textrm{Pr}\left[ W_{(\psi )}\ge k\right] =1-\textrm{Pr}\left[ W_i<k\right] ^\psi =1-(1-\textrm{Pr}\left[ W_i\ge k\right] )^\psi$. Given that the $W_i$ random variables are sampled from a geometric distribution with probability *p*, $\textrm{Pr}\left[ W_i\ge k\right] =(1-p)^k$. Therefore,

$$\begin{aligned} \textrm{E}\mathord {\left[ W_{(\psi )}\right] } = \sum _{k=1}^{\infty }k\left( \left( 1-(1-p)^{k+1}\right) ^\psi -\left( 1-(1-p)^{k}\right) ^\psi \right) . \end{aligned}$$

Now we only take into account the first $x := \lceil \log _{\frac{1}{1-p}} 2 \psi \rceil -1$ summands. Therefore, this bound also holds when conditioning on all $W_i\le 4 \log _{\frac{1}{1-p}} \psi$ if $\psi >1$.

$$\begin{aligned} \textrm{E}\mathord {\left[ W_{(\psi )}\right] }&\ge \sum _{k=1}^{x}k\left( \left( 1-(1-p)^{k+1}\right) ^\psi -\left( 1-(1-p)^{k}\right) ^\psi \right) \\&=\left( 1-(1-p)^{2}\right) ^\psi -\left( 1-(1-p)\right) ^\psi \\&+2\left( 1-(1-p)^{3}\right) ^\psi -2\left( 1-(1-p)^{2}\right) ^\psi \\&+\dots +x\left( 1-(1-p)^{x+1}\right) ^\psi -x\left( 1-(1-p)^{x}\right) ^\psi \\&=\sum _{k=1}^{x}\left( 1-(1-p)^{x+1}\right) ^\psi -\left( 1-(1-p)^{k}\right) ^\psi . \end{aligned}$$

Now we consider only the first $y:=\lfloor \log _{\frac{1}{1-p}} \psi \rfloor$ summands.

$$\begin{aligned} \textrm{E}\mathord {\left[ W_{(\psi )}\right] }&\ge \sum _{k=1}^{y}\left( 1-(1-p)^{x+1}\right) ^\psi -\left( 1-(1-p)^{k}\right) ^\psi \\&\ge y \left( \left( 1-(1-p)^{x+1}\right) ^\psi -\left( 1-(1-p)^{y}\right) ^\psi \right) \\&= \left\lfloor \log _{\frac{1}{1-p}} \psi \right\rfloor \left( \left( 1-(1-p)^{\lceil \log _{\frac{1}{1-p}} 2 \psi \rceil }\right) ^\psi -\left( 1-(1-p)^{\left\lfloor \log _{\frac{1}{1-p}} \psi \right\rfloor }\right) ^\psi \right) \\&\ge \left\lfloor \log _{\frac{1}{1-p}} \psi \right\rfloor \left( \left( 1-\frac{1/2}{\psi }\right) ^\psi -\left( 1-\frac{1}{\psi }\right) ^\psi \right) . \end{aligned}$$

Finally, using $(1 - x)^r \ge 1 - rx$ for $x\ge -1$, $r\in \mathbb {R} \setminus (0,1)$ and $\left( 1-\frac{1}{n}\right) ^n\le e^{-1}$ for $n\ge 1$ we obtain,

$$\begin{aligned} \textrm{E}\mathord {\left[ W_{(\psi )}\right] }&\ge \left( 1/2-e^{-1}\right) \left\lfloor \log _{\frac{1}{1-p}} \psi \right\rfloor . \end{aligned}$$

We now proceed to compute the upper bound. Since the random variable $W_{(\psi )}$ is a non-negative integer, therefore we can also compute its expectation by

$$\begin{aligned} \textrm{E}\mathord {\left[ W_{(\psi )}\right] } = \sum _{k=0}^{\infty }\textrm{Pr}\left[ W_{(\psi )}>k\right] = \sum _{k=1}^{\infty }\textrm{Pr}\left[ W_{(\psi )}\ge k\right] . \end{aligned}$$

As mentioned before $\textrm{Pr}\left[ W_{(\psi )}\ge k\right] =1-(1-(1-p)^k)^\psi$. Therefore,

7 $$\begin{aligned} \textrm{E}\mathord {\left[ W_{(\psi )}\right] } = \sum _{k=1}^{\infty } 1-(1-(1-p)^k)^\psi . \end{aligned}$$

The upper bound is divided in two cases: $\psi =1$ and $\psi >1$. We assume $\psi >1$ first and deal with the other case later. Let us assume that the first $\Big \lceil \log _{\frac{1}{1-p}} \psi \Big \rceil$ summands are 1 then,

$$\begin{aligned} \textrm{E}\mathord {\left[ W_{(\psi )}\right] }&\le \Big \lceil \log _{\frac{1}{1-p}} \psi \Big \rceil + \sum _{k=\Big \lceil \log _{\frac{1}{1-p}} \psi \Big \rceil +1}^{\infty } 1-(1-(1-p)^k)^\psi \\&\le \Big \lceil \log _{\frac{1}{1-p}} \psi \Big \rceil + \sum _{k=1}^{\infty } 1-\left( 1-(1-p)^k (1-p)^{\log _{\frac{1}{1-p}} \psi }\right) ^\psi \\&= \Big \lceil \log _{\frac{1}{1-p}} \psi \Big \rceil + \sum _{k=1}^{\infty } 1-\left( 1-\frac{(1-p)^k}{\psi }\right) ^\psi . \end{aligned}$$

Using $(1 - x)^r \ge 1 - rx$ for $x\ge -1$, $r\in \mathbb {R} {\setminus } (0,1)$ we obtain,

8 $$\begin{aligned} \textrm{E}\mathord {\left[ W_{(\psi )}\right] }&\le \Big \lceil \log _{\frac{1}{1-p}} \psi \Big \rceil + \sum _{k=1}^{\infty } (1-p)^k \nonumber \\&=\Big \lceil \log _{\frac{1}{1-p}} \psi \Big \rceil +\frac{1-p}{p} . \end{aligned}$$

We note that if $\psi =1$ then $\textrm{E}\mathord {\left[ W_{(\psi )}\right] }=\textrm{E}\mathord {\left[ W_i\right] }=\frac{1-p}{p}$. Therefore Eq. (8) holds for this case too.

To compute a bound on $\textrm{E}\mathord {\left[ W_{(\psi )}\mid W_{(\psi )}>j\right] }$ we rely on the *forgetfulness* of the geometric distribution, that is, for $W_i\sim \mathcal {G}(p)$, $\textrm{E}\mathord {\left[ W_i\mid W_i\ge j\right] }= j+\textrm{E}\mathord {\left[ W_i\right] }$. Using the forgetfulness and assuming that all $W_i$ exceed *j* we obtain the simple bound of $\textrm{E}\mathord {\left[ W_{(\psi )}\mid W_{(\psi )}\ge j\right] }=j+\textrm{E}\mathord {\left[ W_{(\psi )}\right] }$. We can improve this bound by noting that only the random variables $W_i$ that are at least *j* increase the expected value. The number of $W_i$ that are at least *j* is in itself a random variable that we call *M*. *M* is sampled from a binomial distribution with parameters $\psi$ and $(1-p)^{j}$, because $\textrm{Pr}\left[ W_i\ge j\right] =(1-p)^{j}$. In addition $M\ge 1$ because at least one $W_i$ has a value at least *j*. Therefore,

$$\begin{aligned} \textrm{E}\mathord {\left[ W_{(\psi )}\mid W_{(\psi )}\ge j\right] }&=j+\textrm{E}\mathord {\left[ \textrm{E}\mathord {\left[ W_{(m)}\mid M=m \wedge M\ge 1\right] }\right] }. \end{aligned}$$

By [30, Lemma 1.8.9] $M\prec (M\mid M\ge 1) \prec (M+1)$, hence,

$$\begin{aligned} \textrm{E}\mathord {\left[ W_{(\psi )}\mid W_{(\psi )}\ge j\right] }&\le j+\textrm{E}\mathord {\left[ \textrm{E}\mathord {\left[ W_{(m+1)}\mid M=m\right] }\right] }\\&= j+\textrm{E}\mathord {\left[ W_{(\psi (1-p)^{j}+1)}\right] }. \end{aligned}$$

$$\square $$

We will also require to understand how reducing the number of random variables affects the expected value of their maximum.

### Lemma 4.2

Let $2\le \psi \in \mathbb {N}$, $0<p<1$ be a constant with respect to $\psi$, $1<c < \psi$, $W_i\sim \mathcal {G}(p)$, $W_{(\psi )}:=\displaystyle \max _{i\in [\psi ]}\{W_i\}$ and $W_{(\psi /c)}:=\displaystyle \max _{i\in [\psi /c]}\{W_i\}$ then,

9 $$\begin{aligned} \textrm{E}\mathord {\left[ W_{(\psi )}\right] }-\textrm{E}\mathord {\left[ W_{(\psi /c)}\right] }&= \Omega (\log \psi ). \end{aligned}$$

### Proof

By definition (cf. Eq. 7) we have

$$\begin{aligned}&\textrm{E}\mathord {\left[ W_{(\psi )}\right] }-\textrm{E}\mathord {\left[ W_{(\psi /c)}\right] } \\&\quad = \sum _{k=1}^{\infty } \left( 1-\left( 1-p\right) ^k\right) ^{\psi /c} - \left( 1-\left( 1-p\right) ^k\right) ^{\psi }\\&\quad \ge \sum _{k=1}^{\log _{\frac{1}{1-p}} \psi /c} \left( 1-\left( 1-p\right) ^k\right) ^{\psi /c} - \left( 1-\left( 1-p\right) ^k\right) ^{\psi }\\&\quad \ge \sum _{k=1}^{\log _{\frac{1}{1-p}} \psi /c} \left( 1-\left( 1-p\right) ^{\log _{\frac{1}{1-p}} \psi /c}\right) ^{\psi /c} - \left( 1-\left( 1-p\right) ^{\log _{\frac{1}{1-p}} \psi /c}\right) ^{\psi }\\&\quad = \sum _{k=1}^{\log _{\frac{1}{1-p}} \psi /c} \left( 1-\frac{c}{\psi }\right) ^{\psi /c} - \left( 1-\frac{c}{\psi }\right) ^{\psi } \end{aligned}$$

Given that $1<c< \psi$ and $\psi \ge 2$, then $0<\left( 1-\frac{c}{\psi }\right) <1$. Since the first exponent $\psi /c$ is smaller than $\psi$ every summand is a positive constant, yielding $\textrm{E}\mathord {\left[ W_{(\psi )}\right] }-\textrm{E}\mathord {\left[ W_{(\psi /c)}\right] }=\Omega (\log \psi )$. $\square$

The expected value is not sufficient to fully understand the behaviour of the algorithm. In the following we show tail bounds on the value that the maximum of several geometric random variables can take.

### Lemma 4.3

Let $0<p<1$ be constant. Let $\delta > 0$, $k\in \mathbb {N}$, $\psi \in \mathbb {N}$, $W_i\sim \mathcal {G}(p)$ and $W_{(\psi )}:=\displaystyle \max _{i\in [\psi ]}\{W_i\}$ then

10 $$\begin{aligned} \textrm{Pr}\left[ W_{(\psi )}\ge \log _{\frac{1}{1-p}}\delta \psi \right]&\le 1/\delta \end{aligned}$$

11 $$\begin{aligned} \textrm{Pr}\left[ W_{(\psi )} > k \mid W_{(\psi )}\le 2 k\right]&\ge 1- \left( \frac{1}{1+p(1-p)^{k+1}}\right) ^{\psi }. \end{aligned}$$

If in addition $k<\textrm{E}\mathord {\left[ W_{(\psi )}\right] }$ then

12 $$\begin{aligned} \textrm{Pr}\left[ W_{(\psi )} > k \mid W_{(\psi )}\le 2 k\right]&\ge 1-\frac{1}{1+p(1-p)^{\frac{1+p}{p}}}. \end{aligned}$$

### Proof

Given that $W_i\sim \mathcal {G}(p)$, $\textrm{Pr}\left[ W_i\right] \ge k$ is the probability that the first *k* Bernoulli trials are unsuccessful, that is $\textrm{Pr}\left[ W_i\ge k\right] =(1-p)^k$. Then we have

13 $$\begin{aligned} \textrm{Pr}\left[ W_{(\psi )}\ge k\right]&=1-\textrm{Pr}\left[ W_i<k\right] ^\psi \nonumber \\&= 1-(1-\textrm{Pr}\left[ W_i\ge k\right] )^\psi \nonumber \\&=1-(1-(1-p)^k)^\psi . \end{aligned}$$

For Eq. (10), let $k=\log _{1/(1-p)}\delta \psi$, and noting that $W_{(\psi )}$ can only be an integer then Eq. (13) yields,

$$\begin{aligned} \textrm{Pr}\left[ W_{(\psi )}\ge k\right]&= \textrm{Pr}\left[ W_{(\psi )}\ge \lceil k\rceil \right] \\&=1-\left( 1-(1-p)^{\Big \lceil \log _{\frac{1}{1-p}}\delta \psi \Big \rceil }\right) ^\psi \\&\le 1-\left( 1-(1-p)^{\log _{\frac{1}{1-p}}\delta \psi }\right) ^\psi =1-\left( 1-\frac{1/\delta }{\psi }\right) ^\psi . \end{aligned}$$

Using $\left( 1+\frac{x}{n}\right) ^n \ge 1+x$ yields the second statement.

To show Eq. (11) we have

$$\begin{aligned} \textrm{Pr}\left[ W_{(\psi )} > k \mid W_{(\psi )}\le 2 k\right]&= 1- \textrm{Pr}\left[ W_{(\psi )} \le k \mid W_{(\psi )}\le 2 k\right] \\&= 1- \textrm{Pr}\left[ W_i \le k \mid W_i\le 2 k\right] ^{\psi }\\&= 1- \left( \frac{1-(1-p)^{k+1}}{1-(1-p)^{2k+1}}\right) ^{\psi }\\&= 1- \left( \frac{1}{\frac{1-(1-p)^{2k+2}-(1-p)^{2k+1}+(1-p)^{2k+2}}{1-(1-p)^{k+1}}}\right) ^{\psi }\\&= 1- \left( \frac{1}{\frac{(1-(1-p)^{k+1})(1+(1-p)^{k+1})-(1-p)^{2k+1}+(1-p)^{2k+2}}{1-(1-p)^{k+1}}}\right) ^{\psi }\\&= 1- \left( \frac{1}{1+(1-p)^{k+1}-(1-p)^{2k+1}\left( \frac{1-(1-p)}{1-(1-p)^{k+1}}\right) }\right) ^{\psi }\\&\ge 1- \left( \frac{1}{1+(1-p)^{k+1}-(1-p)^{2k+1}}\right) ^{\psi }\\&= 1- \left( \frac{1}{1+(1-p)^{k+1}(1-(1-p)^{k})}\right) ^{\psi }\\&\ge 1- \left( \frac{1}{1+p(1-p)^{k+1}}\right) ^{\psi }. \end{aligned}$$

Finally, to show Eq. (12) we note that by Lemma 4.1$k<\textrm{E}\mathord {\left[ W_{(\psi )}\right] }\le \log _{\frac{1}{1-p}} \psi +\frac{1}{p}$, then

$$\begin{aligned} \textrm{Pr}\left[ W_{(\psi )} > k \mid W_{(\psi )}\le 2 k\right]&\ge 1- \left( \frac{1}{1+p(1-p)^{\log _{\frac{1}{1-p}} \psi +\frac{1+p}{p}}}\right) ^{\psi }\\&= 1- \frac{1}{\left( 1+\frac{p(1-p)^{\frac{1+p}{p}}}{\psi }\right) ^{\psi }}\\&\ge 1- \frac{1}{1+p(1-p)^{\frac{1+p}{p}}}. \end{aligned}$$

$$\square $$

Additionally, in Sect. 3 we have seen that the fitness measure $f^{\textrm{avg}}$ assigns fitness to an individual based on the sum of distances to $\alpha$ or $\beta$. The following lemma is useful to understand how much this sum can change by this effect.

### Lemma 4.4

Let $h\in \mathbb {N}$ and $W_i$ be i.i.d. random variables sampled from $\mathcal {G}(p)$. Then,

$$\begin{aligned} \textrm{Pr}\left[ \sum _{i=1}^{\psi }(W_i+h)-\sum _{i=\psi +1}^{2\psi }(W_i-h)\le 0\right] =2\exp {\left( -\frac{h^2p^2\psi }{2(1-p)^2}\right) }. \end{aligned}$$

Before showing Lemma 4.4 we need the following helper lemma.

### Lemma 4.5

Let $0<p<1$ be a constant, $h\in \mathbb {N}$, $\psi \in \mathbb {N}$, $W_i\sim \mathcal {G}(p)$ and $W:=\sum _{i=1}^{\psi } W_i$ then, there exists a constant $c<1$ for which the following statements are simultaneously true

$$\begin{aligned} \textrm{Pr}\left[ W \le \left( \frac{c(1-p)}{p} - h\right) \psi \right]&\le \exp {\left( -\frac{h^2p^2\psi }{2(1-p)^2}\right) },\\ \textrm{Pr}\left[ W \ge \left( \frac{c(1-p)}{p} + h\right) \psi \right]&\le \exp {\left( -\frac{h^2p^2\psi }{2(1-p)^2}\right) }. \end{aligned}$$

### Proof

If $h>\frac{c(1-p)}{p}$ then $\left( \frac{c(1-p)}{p} - h\right) <0$ and the first probability is 0. Otherwise, we note that for $\delta :=1-c+\frac{hp}{1-p}$

$$\begin{aligned} \textrm{Pr}\left[ W \le \left( \frac{c(1-p)}{p} - h\right) \psi \right]&= \textrm{Pr}\left[ W \le (1-\delta ) \frac{1-p}{p} \psi \right] . \end{aligned}$$

Then, using Chernoff bounds for geometric random variables [30, Theorem 1.10.32] we obtain

$$\begin{aligned} \textrm{Pr}\left[ W \le (1-\delta ) \frac{(1-p)}{p} \psi \right]&\le \exp {\left( -\frac{\delta ^2 \psi }{2-\frac{4\delta }{3}}\right) } \\&\le \exp {\left( -\frac{\delta ^2 \psi }{2}\right) } \\&\le \exp {\left( -\left( \frac{hp}{1-p}\right) ^2\frac{\psi }{2}\right) } \\&= \exp {\left( -\frac{h^2p^2\psi }{2(1-p)^2}\right) } \\ \end{aligned}$$

The last inequality holds for all constant $0\le c\le 1$.

For the second statement, let $\delta :=\frac{hp}{1-p}-1+c$, then for $c:=\max \left\{ \frac{2-3p}{2(1-p)}, 0\right\}$ and all $h\in \mathbb {N}$ it holds that $\delta >0$ and

$$\begin{aligned} \textrm{Pr}\left[ W \ge \left( \frac{c(1-p)}{p}+h\right) \psi \right]&=\textrm{Pr}\left[ W \ge \left( \frac{hp}{1-p}+c\right) \frac{1-p}{p} \psi \right] \\&=\textrm{Pr}\left[ W \ge \left( 1+\delta \right) \frac{1-p}{p} \psi \right] . \end{aligned}$$

Finally, using Chernoff bounds for geometric random variables [30, Theorem 1.10.32] we obtain

$$\begin{aligned} \textrm{Pr}\left[ W \ge \left( 1+\delta \right) \frac{1-p}{p} \psi \right]&\le \exp {\left( -\frac{\delta ^2 (\psi -1)}{2(1+\delta )}\right) }\\&\le \exp {\left( -\frac{\left( \frac{(2h-1)p}{2(1-p)}\right) ^2 (\psi -1)}{2\left( \frac{2+(2h-3)p}{2(1-p)}\right) }\right) }\\&= \exp {\left( -\frac{(2h-1)^2p^2 (\psi -1)}{4(1-p)(2+(2h-3)p)}\right) }\\&\le \exp {\left( -\frac{h^2p^2\psi }{2(1-p)^2}\right) } \end{aligned}$$

Noting that for all constant $0<p<1$, $\frac{2-3p}{2(1-p)}<1$ shows that there exists a $c<1$ for which the above holds. $\square$

With Lemma 4.5 we can prove Lemma 4.4.

### Proof of Lemma 4.4

Let

$$\begin{aligned} Y:=\sum _{i=1}^{\psi }(W_i+h)-\sum _{i=\psi +1}^{2\psi }(W_i-h) = 2 \psi h + \sum _{i=1}^{\psi } W_i -\sum _{i=\psi +1}^{2\psi } W_i. \end{aligned}$$

We pessimistically assume that if $\sum _{i=1}^{\psi }W_i\le \left( \frac{c(1-p)}{p}-h\right) \psi$ (for some $c<1$ that meets the condition of Lemma 4.5) then $Y\le 0$. By Lemma 4.5 the probability of this event is at most $\exp {\left( -\frac{h^2p^2\psi }{2(1-p)^2}\right) }$. Otherwise,

$$\begin{aligned} 2\psi h + \sum _{i=1}^{\psi } W_i -\sum _{i=\psi +1}^{2\psi } W_i \ge \psi \left( \frac{c(1-p)}{p} + h\right) +1-\sum _{i=\psi +1}^{2\psi } W_i. \end{aligned}$$

By Lemma 4.5 the probability that $\sum _{i=\psi +1}^{2\psi } W_i\ge \psi \left( \frac{c(1-p)}{p} + h\right)$ is at most $\exp {\left( -\frac{h^2p^2\psi }{2(1-p)^2}\right) }$. A union bound of both events shows that $\textrm{Pr}\left[ Y\le 0\right] =2\exp {\left( -\frac{h^2p^2\psi }{2(1-p)^2}\right) }$. $\square$

## Averaging Interactions is Inefficient

In this section we study the fitness measure $f^{\textrm{avg}}$. We show that the ${(1 , \lambda )}~\textrm{CoEA}$ using $f^{\textrm{avg}}$ is inefficient on Bilinear. Particularly we show that it has an expected runtime that is infinite, this is shown in Theorem 5.1.

### Theorem 5.1

Consider the ${(1 , \lambda )}~\textrm{CoEA}$ using $f^{\textrm{avg}}$. Let $M_t:=\left|x_{t}-\beta \right|+\left|y_t-\alpha \right|$ be the Manhattan distance to the optimum and the initial distance ${M_0>0}$. Define $T=\inf \{t\mid M_t=0\}$. If $\lambda \ge 2 (1-p)^{-8}$ and the following condition holds:

14 $$\begin{aligned}&2\exp {\left( -\frac{p^2 \lambda }{4(1-p)^2}\right) } \cdot \left( \Big \lceil \log _{\frac{1}{1-p}} \lambda /2\Big \rceil +\frac{1-p}{p}\right) \nonumber \\&\qquad \qquad \qquad <\left( 1-2\exp {\left( -\frac{p^2 \lambda }{4(1-p)^2}\right) }\right) \cdot \left( 1- \frac{1}{1+p(1-p)^{\frac{1+p}{p}}}\right) \end{aligned}$$

then, $$\textrm{E}\mathord {\left[ T\right] }=\infty $$.

We note that the conditions in Theorem 5.1 depend only in $\lambda$ and *p*. In addition, the left-hand side of (14) goes to zero as $\lambda \rightarrow \infty$ and the right-hand side is monotonically increasing in $\lambda$. Therefore, for a sufficiently large $\lambda$ the condition holds. Via numerical methods we have determined that $\lambda >(3/p)^3$ meets the condition. In Table 1 we show the minimum $\lambda$-value that meets the condition for different *p* and note that $p=0.5$ yields an expected distance of 1 between the offspring and their parent.

**Table 1:** Minimum $\lambda$-value that meets condition (14) for different *p*

p	0.1	0.2	0.3	0.4	0.5	0.6	0.7
$\lambda$	2768	452	135	51	21	9	4

**Fig. 3:**
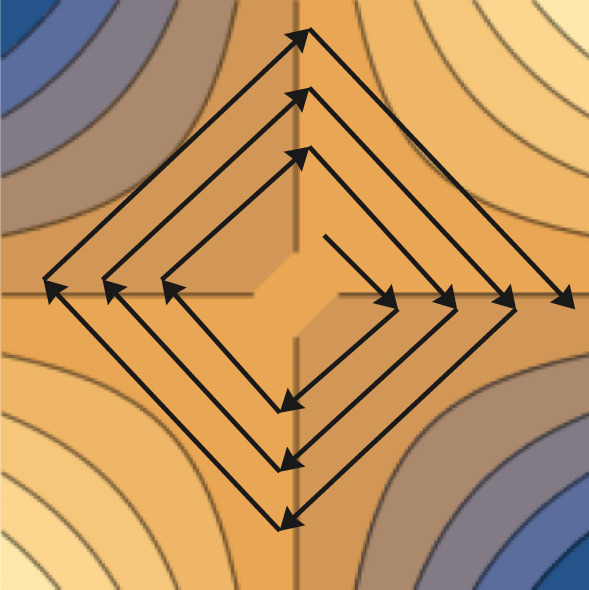
Behaviour of a typical run of ${(1 , \lambda )}~\textrm{CoEA}$ with fitness measure $f^{\textrm{avg}}$ on Bilinear

The main challenge of the proof is to show that at every generation the algorithm moves away from the optimum in expectation. Outside from the technicalities of the proof the main reason this happens is that in a typical run the algorithm will tend to stay at the same distance to the optimum. Although the distance to the optimum tend to stay the same, the algorithm is continuously moving in a clockwise manner throughout the search space. The generations where the algorithm clearly moves away from the optimum are the generations where the algorithm transitions from one quadrant to the other. This is because in these generations the parent is in the previous quadrant and the majority of the offspring tends to also be in the previous quadrant, therefore the highest fitness is assigned as if the algorithm stayed in the same quadrant and the algorithm moves away from the optimum with high probability. Figure 3 visualises this behaviour.

Although typically the algorithm does not move away from the optimum when it is far from another quadrant (which is the majority of generations) there is always a non-negative probability of that event happening which result in a (sometimes tiny) negative *drift* everywhere. We compute this change in distance to the optimum in the next lemma.

### Lemma 5.2

Consider the ${(1 , \lambda )}~\textrm{CoEA}$ using $f^{\textrm{avg}}$. Let $M_t:=\left|x_{t}-\beta \right|+\left|y_t-\alpha \right|$ be the Manhattan distance to the optimum. $\textrm{E}_t\mathord {\left[ M_{t+1}-M_t\right] }>0$ provided that $\lambda \ge 2 (1-p)^{-8}$ and the following condition holds:

15 $$\begin{aligned}&2\exp {\left( -\frac{p^2 \lambda }{4(1-p)^2}\right) } \cdot \left( \Big \lceil \log _{\frac{1}{1-p}} \lambda /2\Big \rceil +\frac{1-p}{p}\right) \nonumber \\&\qquad \qquad \qquad <\left( 1-2\exp {\left( -\frac{p^2 \lambda }{4(1-p)^2}\right) }\right) \cdot \left( 1- \frac{1}{1+p(1-p)^{\frac{1+p}{p}}}\right) \end{aligned}$$

### Proof

To simplify things we will assume that at time *t* the current parents $x_t, y_t$ reside in the second quadrant, that is, $x_t\ge \beta$ and $y_t>\alpha$, and due to the symmetry of the search space, the computations translate to other quadrants.

At each step the algorithm assigns the highest fitness to individuals in $P_t$ ($Q_t$) depending on $Y_t=\sum _{y\in Q_t}\left( y-\alpha \right)$ ($X_t=\sum _{x\in P_t}\left( x-\beta \right)$), that is the sum of distances to the optimum of the competing population $Q_t$ ($P_t$). Therefore, there are three different cases for $X_t<0,X_t=0,X_t>0$ and combining these with $Y_t<0,Y_t=0,Y_t>0$ gives nine different cases in total. We will deal with each case separately.

(i) ($X_t<0$,$Y_t<0$). In this case, by Lemma 3.1 the algorithm will select $x_{t+1}=x_{\min }$ and $y_{t+1}=y_{\max }$. Recall that the algorithm creates $\lambda /2$ offspring with $x\ge x_t\ge \beta$ and $\lambda /2$ offspring with $x\le x_t$. If $X_t<0$ there exists at least one offspring *x* where $\beta -x > x_t-\beta$ and $\left|x-\beta \right|>\left|x_{t}-\beta \right|$, that is $\left|x_{t+1}-\beta \right|>\left|x_{t}-\beta \right|$. Similarly the algorithm creates $\lambda /2$ offspring with $y\ge y_t>\alpha$ therefore $Y_t<0$ implies $\left|y_{t+1}-\alpha \right|> \left|y_{t}-\alpha \right|$. This yields $M_{t+1}-M_t\ge 2$.
(ii) ($X_t<0$,$Y_t=0$). In this case the algorithm will select $x_{t+1}$ u. a. r. from $P_t$ and $y_{t+1}=y_{\max }$. Let $x^{(i)}$ be the offspring $i\in [\lambda ]$ of $x_t$. Then as before, the algorithm creates $x^{(i)}\ge x_t\ge \beta$ for $i\in \{1,\dots , \lambda /2\}$ and $x^{(i)}\le x_t$ for $i\in \{\lambda /2+1,\dots , \lambda \}$. Therefore, $X_t<0$ implies
  $$\begin{aligned} \sum _{i=1}^{\lambda /2} \left( x^{(i)} - \beta \right) < \sum _{i=\lambda /2+1}^{\lambda } \left( \beta - x^{(i)}\right) . \end{aligned}$$
  Additionally,
  $$\begin{aligned} \sum _{i=1}^{\lambda /2} \left( x^{(i)} - \beta \right) \ge \frac{\lambda }{2}\left( x_t - \beta \right) . \end{aligned}$$
  Since $$\beta \in \mathbb {Z} $$, then
  $$\begin{aligned} \frac{\sum _{i=1}^{\lambda } \left|x^{(i)} - \beta \right|}{\lambda } {\ge \frac{\lambda (x_t - \beta )+1}{\lambda }} =x_t - \beta +\frac{1}{\lambda }. \end{aligned}$$
  Therefore in expectation the offspring chosen from $P_t$ is at least $1/\lambda$ farther from the optimum. For $y_{t+1}$, as before, the algorithm creates $\lambda /2$ offspring with $y\ge y_t>\alpha$ therefore $Y_t=0$ implies $\left|y_{t+1}-\alpha \right|\ge \left|y_{t}-\alpha \right|$. Both changes result in
  $$\begin{aligned} \textrm{E}_t\mathord {\left[ M_{t+1}-M_t\mid X_t<0,Y_t=0\right] }\ge 1/\lambda >0. \end{aligned}$$
(iii) ($X_t<0$,$Y_t>0$). In this case the algorithm will select $x_{t+1}=x_{\max }$ and $y_{t+1}=y_{\max }$. Considering the tendency for event $X_t < 0$ to coincide with smaller values of $x_{max}\ge x_t$, we adopt a pessimistic assumption that $x_{t+1} = x_t$. In the case of $y_{t+1}$, $Y_t>0$ increases the probability of higher values of $y_{\max }$, therefore $\textrm{E}_t\mathord {\left[ y_{\max }\mid Y_t>0\right] }\ge \textrm{E}_t\mathord {\left[ y_{\max }\right] }$. Then, $\textrm{E}_t\mathord {\left[ y_{\max }\mid X_t<0,Y_t>0\right] }\ge y_t+\textrm{E}\mathord {\left[ W_{(\lambda /2)}\right] }$, where $W_{(\lambda /2)}$ is the maximum value of $\lambda /2$ geometrically distributed independent random variables. By Lemma 4.1 this is at least $\left( 1/2-e^{-1}\right) \lfloor \log _{\frac{1}{1-p}} (\lambda /2) \rfloor$. Hence,
  $$\begin{aligned} \textrm{E}_t\mathord {\left[ M_{t+1}-M_t\mid X_t<0,Y_t>0\right] }\ge \left( 1/2-e^{-1}\right) \lfloor \log _{\frac{1}{1-p}} (\lambda /2) \rfloor \ge 0. \end{aligned}$$
(iv) ($X_t=0$,$Y_t<0$). In this case the algorithm will select $x_{t+1}=x_{\min }$ and $y_{t+1}$ u. a. r. from $Q_t$. Similar to $X_t<0$, $X_t=0$ implies that $\left|x_{\min }-\beta \right|\ge \left|x_{t}-\beta \right|$. And similar to case (ii) $Y_t<0$ imply that in expectation the offspring chosen from $Q_t$ is at least $1/\lambda$ farther from the optimum than its parent. Therefore,
  $$\begin{aligned} \textrm{E}_t\mathord {\left[ M_{t+1}-M_t\mid X_t=0,Y_t<0\right] }\ge 1/\lambda > 0. \end{aligned}$$
(v) ($X_t=0$,$Y_t=0$). In this case the algorithm will select $x_{t+1}$ u. a. r. from $P_t$ and $y_{t+1}$ u. a. r. from $Q_t$. Similar to (ii) and (iv) $X_t=0$ and $Y_t=0$ imply that in expectation the offspring chosen are at least as far from the optimum than its parents. Then,
  $$\begin{aligned} \textrm{E}_t\mathord {\left[ M_{t+1}-M_t\mid X_t=0,Y_t=0\right] }\ge 0. \end{aligned}$$
(vi) ($X_t=0$,$Y_t>0$). In this case the algorithm will select $x_{t+1}=x_{\max }$ and $y_{t+1}$ u. a. r. from $Q_t$. The event $X_t=0$ is negatively correlated with the value of $x_{\max }\ge x_t$, therefore we pessimistically assume that $x_{t+1}=x_t$. For $y_{t+1}$, we recall that $Y_t= \sum _{y\in Q_t}\left( y-\alpha \right)$, therefore if $Y_t>0$, then for all offspring *y* we have $\textrm{E}\mathord {\left[ y\mid Y_t>0\right] }\ge \textrm{E}\mathord {\left[ y\right] }$. Since we select an offspring u. a. r., in expectation $y_{t+1}$ will be at least as large as $y_{t}$ and $\left|y_{t+1}-\alpha \right|\ge \left|y_{t}-\alpha \right|$ giving
  $$\begin{aligned} \textrm{E}_t\mathord {\left[ M_{t+1}-M_t\mid X_t=0,Y_t>0\right] }\ge 0. \end{aligned}$$
(vii) ($X_t>0$,$Y_t<0$). In this case the algorithm will select $x_{t+1}=x_{\min }$ and $y_{t+1}=y_{\min }$. The change in distance $\left|x_{t+1}-\beta \right|-\left|x_{t}-\beta \right|$ depends on whether $x_{\min }<\beta$ or $x_{\min }\ge \beta$. Let $\Delta _x:=x_{t+1}-x_{t}$. If $x_{\min }<\beta$ then
  $$\begin{aligned} \left|x_{t+1}-\beta \right|-\left|x_{t}-\beta \right|&= (\beta -x_{t+1})-(x_{t}-\beta )\\&= 2\beta +x_{t+1}-x_{t}-2 x_{t+1}\\&= 2(\beta -x_{t+1})+\Delta _x \ge \Delta _x. \end{aligned}$$
  Otherwise
  $$\begin{aligned} \left|x_{t+1}-\beta \right|-\left|x_{t}-\beta \right| = (x_{t+1}-\beta )-(x_{t}-\beta ) = x_{t+1}-x_{t} = \Delta _x. \end{aligned}$$
  Therefore, we pessimistically assume that $x_{\min }\ge \beta$. Given this assumption $X_t>0$ is positively correlated with the change in distance and we can bound the conditional expectation with the unconditional expectation. And similar to case (i) $Y_t<0$ imply that the offspring chosen from $Q_t$ is at least 1 farther from the optimum than its parent. Hence,
  $$\begin{aligned} \textrm{E}_t\mathord {\left[ M_{t+1}-M_t\mid X_t>0,Y_t<0\right] }&\ge 1 + \textrm{E}_t\mathord {\left[ x_{t+1}-x_{t}\mid X_t>0,Y_t<0\right] } \\&\ge 1- \textrm{E}\mathord {\left[ W_{(\lambda /2)}\right] }. \end{aligned}$$
(viii) ($X_t>0$,$Y_t=0$). In this case the algorithm will select $x_{t+1}$ u. a. r. from $P_t$ and $y_{t+1}=y_{\min }$. With the same arguments as in (vi) we can see that in expectation $x_{t+1}\ge x_{t}$. For $y_{t+1}$ as in (iv) and (v) $Y_t=0$ implies that $\left|y_{\min }-\alpha \right|\ge \left|y_{t}-\alpha \right|$. Therefore,
  $$\begin{aligned} \textrm{E}_t\mathord {\left[ M_{t+1}-M_t\mid X_t>0,Y_t=0\right] }&\ge 0. \end{aligned}$$
(ix) ($X_t>0$,$Y_t>0$). We note that this is the most probable case at every generation of the algorithm when in the second quadrant. In this case the algorithm will select $x_{t+1}=x_{\max }$ and $y_{t+1}=y_{\min }$. The event $X_t>0$ is positively correlated with $x_{\max }$ and since $x_{\max }\ge x_{t}\ge \beta$
  16 $$\begin{aligned} \textrm{E}_t\mathord {\left[ \left|x_{t+1}-\beta \right|-\left|x_{t}-\beta \right|\mid X_t>0,Y_t>0\right] }&=\textrm{E}_t\mathord {\left[ x_{t+1}-x_{t}\mid X_t>0,Y_t>0\right] }\nonumber \\&\ge \textrm{E}\mathord {\left[ W_{(\lambda /2)}\right] }. \end{aligned}$$
  For $y_{t+1}$ we will divide it in two more cases, $y_{t}-\alpha \ge \textrm{E}\mathord {\left[ W_{(\lambda /2)}\right] }$ and $y_{t}-\alpha < \textrm{E}\mathord {\left[ W_{(\lambda /2)}\right] }$. For the first case, as in (vii) we pessimistically assume that $y_{t+1}>\alpha$ and
  17 $$\begin{aligned}&\textrm{E}_t\mathord {\left[ \left|y_{t+1}-\alpha \right|-\left|y_{t}-\alpha \right|\mid y_{t}-\alpha \ge \textrm{E}\mathord {\left[ W_{(\lambda /2)}\right] },X_t>0,Y_t>0\right] }\nonumber \\&\quad \ge \textrm{E}_t\mathord {\left[ y_{t+1}-y_{t}\mid Y_t>0\right] }. \end{aligned}$$
  If $y_{t}-\alpha < \textrm{E}\mathord {\left[ W_{(\lambda /2)}\right] }$ and additionally $y_{t+1}\ge \alpha$ we have,
  18 $$\begin{aligned}&\textrm{E}_t\mathord {\left[ \left|y_{t+1}-\alpha \right|-\left|y_{t}-\alpha \right|\mid y_{t}-\alpha< \textrm{E}\mathord {\left[ W_{(\lambda /2)}\right] },X_t>0, Y_t>0, y_{t+1}\ge \alpha \right] }\nonumber \\&\quad =\textrm{E}_t\mathord {\left[ y_{t+1}-y_{t}\mid y_{t}-\alpha < \textrm{E}\mathord {\left[ W_{(\lambda /2)}\right] },Y_t>0, y_{t+1}\ge \alpha \right] }\nonumber \\&\quad \ge \alpha -y_{t}>- \textrm{E}\mathord {\left[ W_{(\lambda /2)}\right] }. \end{aligned}$$
  If instead $y_{t+1}<\alpha$ we have,
  19 $$\begin{aligned}&\textrm{E}_t\mathord {\left[ \left|y_{t+1}-\alpha \right|-\left|y_{t}-\alpha \right|\mid y_{t}-\alpha< \textrm{E}\mathord {\left[ W_{(\lambda /2)}\right] }, X_t>0,Y_t>0,y_{t+1}<\alpha \right] }\nonumber \\&\quad =\textrm{E}_t\mathord {\left[ -y_{t+1}-y_{t}+2\alpha \mid y_{t}-\alpha< \textrm{E}\mathord {\left[ W_{(\lambda /2)}\right] },Y_t>0, y_{t+1}<\alpha \right] }\nonumber \\&\quad \ge -(\alpha -1)-y_{t}+2\alpha =\alpha -y_{t}+1\nonumber \\&\quad >- \textrm{E}\mathord {\left[ W_{(\lambda /2)}\right] }+1. \end{aligned}$$
  Therefore, for case (ix) if $y_t-\alpha \ge \textrm{E}\mathord {\left[ W_{(\lambda /2)}\right] }$, using Eqs. (16) and (17) we have
  $$\begin{aligned}&\textrm{E}_t\mathord {\left[ M_{t+1}-M_t\mid y_t-\alpha \ge \textrm{E}\mathord {\left[ W_{(\lambda /2)}\right] }, X_t>0,Y_t>0\right] }\\&\quad = \textrm{E}_t\mathord {\left[ \left|x_{t+1}-\beta \right|-\left|x_{t}-\beta \right|\mid X_t>0,Y_t>0\right] } + \\&\quad \textrm{E}_t\mathord {\left[ \left|y_{t+1}-\alpha \right|-\left|y_{t}-\alpha \right|\mid y_t-\alpha \ge \textrm{E}\mathord {\left[ W_{(\lambda /2)}\right] }, X_t>0,Y_t>0\right] }\\&\quad \ge \textrm{E}\mathord {\left[ W_{(\lambda /2)}\right] } +\textrm{E}_t\mathord {\left[ y_{t+1}-y_{t}\mid Y_t>0\right] }, \end{aligned}$$
  and if $y_t-\alpha < \textrm{E}\mathord {\left[ W_{(\lambda /2)}\right] }$ with Eq. (16) and combining Eqs. (18) and (19) with the law of total expectation we have
  $$\begin{aligned}&\textrm{E}_t\mathord {\left[ M_{t+1}-M_t\mid y_t-\alpha< \textrm{E}\mathord {\left[ W_{(\lambda /2)}\right] }, X_t>0,Y_t>0\right] }\\&\quad = \textrm{E}_t\mathord {\left[ \left|x_{t+1}-\beta \right|-\left|x_{t}-\beta \right|\mid X_t>0,Y_t>0\right] } + \\&\qquad \textrm{Pr}\left[ y_{t+1}\ge \alpha \mid Y_t>0\right] \cdot \\&\qquad \textrm{E}_t\mathord {\left[ \left|y_{t+1}-\alpha \right|-\left|y_{t}-\alpha \right|\mid y_t-\alpha< \textrm{E}\mathord {\left[ W_{(\lambda /2)}\right] },Y_t>0, y_{t+1}\ge \alpha \right] } + \\&\qquad \textrm{Pr}\left[ y_{t+1}<\alpha \mid Y_t>0\right] \cdot \\&\qquad \textrm{E}_t\mathord {\left[ \left|y_{t+1}-\alpha \right|-\left|y_{t}-\alpha \right|\mid y_t-\alpha< \textrm{E}\mathord {\left[ W_{(\lambda /2)}\right] },Y_t>0, y_{t+1}<\alpha \right] }\\&\quad \ge \textrm{E}\mathord {\left[ W_{(\lambda /2)}\right] } - \textrm{Pr}\left[ y_{t+1}\ge \alpha \mid Y_t>0\right] \textrm{E}\mathord {\left[ W_{(\lambda /2)}\right] }\\&\qquad +\textrm{Pr}\left[ y_{t+1}<\alpha \mid Y_t>0\right] \left( 1-\textrm{E}\mathord {\left[ W_{(\lambda /2)}\right] }\right) . \end{aligned}$$

This finishes the computations of all nine cases.

Now, we put everything together to compute $\textrm{E}_t\mathord {\left[ M_{t+1}-M_t\right] }$. To ease computations and since we want a lower bound, we omit all cases with positive contributions except for case (ix). We keep the only case with a negative contribution, which is case (vii). This yields the following two conditional expectations:

1. If $$y_t-\alpha \ge \textrm{E}\mathord {\left[ W_{(\lambda /2)}\right] }$$
  $$\begin{aligned}&\textrm{E}_t\mathord {\left[ M_{t+1}-M_t\mid y_t-\alpha \ge \textrm{E}\mathord {\left[ W_{(\lambda /2)}\right] }\right] }\\&\quad \ge \textrm{Pr}\left[ X_t>0\right] \left( \textrm{Pr}\left[ Y_t<0\right] \left( 1- \textrm{E}\mathord {\left[ W_{(\lambda /2)}\right] }\right) \right. \\&\quad \left. +\textrm{Pr}\left[ Y_t>0\right] (\textrm{E}\mathord {\left[ W_{(\lambda /2)}\right] }+\textrm{E}_t\mathord {\left[ y_{t+1}-y_{t}\mid Y_t>0\right] })\right) \end{aligned}$$
  using the law of total expectation,
  $$\begin{aligned}&= \textrm{Pr}\left[ X_t>0\right] \bigg (\textrm{Pr}\left[ Y_t<0\right] \left( 1- \textrm{E}\mathord {\left[ W_{(\lambda /2)}\right] }\right) +\textrm{Pr}\left[ Y_t>0\right] \bigg . \\&\quad \left. \cdot \left( \textrm{E}\mathord {\left[ W_{(\lambda /2)}\right] }+\frac{\textrm{E}_t\mathord {\left[ y_{t+1}-y_{t}\right] }-\textrm{Pr}\left[ Y_t\le 0\right] \textrm{E}_t\mathord {\left[ y_{t+1}-y_{t}\mid Y_t\le 0\right] }}{\textrm{Pr}\left[ Y_t>0\right] }\right) \right) \\&\quad = \textrm{Pr}\left[ X_t>0\right] \left( \textrm{Pr}\left[ Y_t<0\right] \left( 1- \textrm{E}\mathord {\left[ W_{(\lambda /2)}\right] }\right) +\textrm{Pr}\left[ Y_t>0\right] \textrm{E}\mathord {\left[ W_{(\lambda /2)}\right] }\right. \\&\quad \left. +\textrm{E}_t\mathord {\left[ y_{t+1}-y_{t}\right] } - \textrm{E}_t\mathord {\left[ y_{t+1}-y_{t}\mid Y_t\le 0\right] }\textrm{Pr}\left[ Y_t\le 0\right] \right) \\&\quad = \textrm{Pr}\left[ X_t>0\right] \left( \textrm{Pr}\left[ Y_t<0\right] \left( 1- \textrm{E}\mathord {\left[ W_{(\lambda /2)}\right] }\right) +\left( 1-\textrm{Pr}\left[ Y_t\le 0\right] \right) \textrm{E}\mathord {\left[ W_{(\lambda /2)}\right] }\right. \\&\quad \left. +\textrm{E}_t\mathord {\left[ y_{t+1}-y_{t}\right] } - \textrm{E}_t\mathord {\left[ y_{t+1}-y_{t}\mid Y_t\le 0\right] }\textrm{Pr}\left[ Y_t\le 0\right] \right) . \end{aligned}$$
  Due to the condition $\lambda \ge 2(1-p)^{-8}$ and Lemma 4.1$1- \textrm{E}\mathord {\left[ W_{(\lambda /2)}\right] }<0$. Then using $\textrm{Pr}\left[ Y_t< 0\right] \le \textrm{Pr}\left[ Y_t\le 0\right]$ and $\textrm{E}_t\mathord {\left[ y_{t+1}-y_{t}\right] }\ge -\textrm{E}\mathord {\left[ W_{(\lambda /2)}\right] }$ we obtain,
  $$\begin{aligned}&\ge \textrm{Pr}\left[ X_t>0\right] \left( \textrm{Pr}\left[ Y_t\le 0\right] \left( 1- 2 \textrm{E}\mathord {\left[ W_{(\lambda /2)}\right] }-\textrm{E}\mathord {\left[ y_{t+1}-y_{t}\mid Y_t\le 0\right] }\right) \right. \\&\quad \left. +\textrm{E}\mathord {\left[ W_{(\lambda /2)}\right] }-\textrm{E}\mathord {\left[ W_{(\lambda /2)}\right] }\right) . \end{aligned}$$
  We note that the algorithm creates half of the offspring in $Q_t$ with a value greater than $y_t$, therefore $\displaystyle \sum _{y\in Q_t}\left( y-\alpha \right) =Y_t\le 0$ imply that $\alpha - y_{\min }\ge y_t - \alpha$ and due to $X_t>0$, $y_{t+1}=y_{\min }$. Hence we can bound $-\textrm{E}\mathord {\left[ y_{t+1}-y_{t}\mid Y_t\le 0\right] }\ge 2(y_t-\alpha )$.
  $$\begin{aligned}&\ge \textrm{Pr}\left[ X_t>0\right] \left( \textrm{Pr}\left[ Y_t\le 0\right] \left( 1- 2 \textrm{E}\mathord {\left[ W_{(\lambda /2)}\right] }+2(y_t-\alpha )\right) \right) \\&\quad \ge \textrm{Pr}\left[ X_t>0\right] \left( \textrm{Pr}\left[ Y_t\le 0\right] \left( 1- 2 \textrm{E}\mathord {\left[ W_{(\lambda /2)}\right] }+2 \textrm{E}\mathord {\left[ W_{(\lambda /2)}\right] }\right) \right) \\&\quad = \textrm{Pr}\left[ X_t>0\right] \textrm{Pr}\left[ Y_t\le 0\right] >0. \end{aligned}$$
2. And if $y_t-\alpha < \textrm{E}\mathord {\left[ W_{(\lambda /2)}\right] }$ then,
  $$\begin{aligned}&\textrm{E}_t\mathord {\left[ M_{t+1}-M_t\mid y_t-\alpha< \textrm{E}\mathord {\left[ W_{(\lambda /2)}\right] }\right] }\\&\quad \ge \textrm{Pr}\left[ X_t>0\right] \left( \textrm{Pr}\left[ Y_t\le 0\right] \left( 1- \textrm{E}\mathord {\left[ W_{(\lambda /2)}\right] }\right) \right. \\&\qquad +\textrm{Pr}\left[ Y_t>0\right] \left( \textrm{E}\mathord {\left[ W_{(\lambda /2)}\right] }-\textrm{Pr}\left[ y_{t+1}\ge \alpha \mid Y_t>0\right] \textrm{E}\mathord {\left[ W_{(\lambda /2)}\right] }\right. \\&\qquad \left. \left. +\,\textrm{Pr}\left[ y_{t+1}<\alpha \mid Y_t>0\right] (1- \textrm{E}\mathord {\left[ W_{(\lambda /2)}\right] })\right) \right) \\&\quad = \textrm{Pr}\left[ X_t>0\right] \left( \textrm{Pr}\left[ Y_t\le 0\right] \left( 1- \textrm{E}\mathord {\left[ W_{(\lambda /2)}\right] }\right) \right. \\&\qquad \left. +\textrm{Pr}\left[ Y_t>0\right] \textrm{Pr}\left[ y_{t+1}<\alpha \mid Y_t>0\right] \right) . \end{aligned}$$
  The probability $\textrm{Pr}\left[ y_{t+1}<\alpha \mid Y_t>0\right]$ is at least
  $$\begin{aligned} \textrm{Pr}\left[ y_{t}-y_{\min }>y_{t}-\alpha \mid y_{t}-y_{\min }\le 2(y_{t}-\alpha )\right] , \end{aligned}$$
  since the condition $Y_t>0$ is met if $y_{t}-y_{\min }\le 2(y_{t}-\alpha )$ and $y_{t+1}=y_{\min }$. In turn, this is equivalent to $\textrm{Pr}\left[ W_{(\lambda /2)} > k \mid W_{(\lambda /2)}\le 2 k\right]$ for $k=y_{t}-\alpha <\textrm{E}\mathord {\left[ W_{(\lambda /2)}\right] }$ and by Lemma 4.3 (12) is at least $1-\frac{1}{1-p(1-p)^{\frac{1+p}{p}}}$. By Lemma 4.4$\textrm{Pr}\left[ Y_t\le 0\right] \le 2\exp {\left( -\frac{p^2\lambda }{4(1-p)^2}\right) }$, $\textrm{Pr}\left[ Y_t>0\right] \ge 1-2\exp {\left( -\frac{p^2\lambda }{4(1-p)^2}\right) }$, $\textrm{Pr}\left[ X_t>0\right] =c$ for some constant $0<c<1$. Additionally, by Lemma 4.1$(1-\textrm{E}\mathord {\left[ W_{(\lambda /2)}\right] })\ge -\Big \lceil \log _{\frac{1}{1-p}} \lambda /2\Big \rceil +\frac{1-p}{p}$. Therefore, we obtain
  $$\begin{aligned}&\textrm{E}_t\mathord {\left[ M_{t+1}-M_t\mid y_t-\alpha < \textrm{E}\mathord {\left[ W_{(\lambda /2)}\right] }\right] }\\&\quad \ge c \cdot \left( \left( 1-2\exp {\left( -\frac{p^2 \lambda }{4(1-p)^2}\right) }\right) \cdot \left( 1- \frac{1}{1+p(1-p)^{\frac{1+p}{p}}}\right) \right. \nonumber \\&\qquad - \left. 2\exp {\left( -\frac{p^2 \lambda }{4(1-p)^2}\right) } \cdot \left( \Big \lceil \log _{\frac{1}{1-p}} \lambda /2\Big \rceil +\frac{1-p}{p}\right) \right) . \end{aligned}$$
  Finally, with the condition on $\lambda$ and *p* in the statement
  $$\begin{aligned}&\textrm{E}_t\mathord {\left[ M_{t+1}-M_t\mid y_t-\alpha < \textrm{E}\mathord {\left[ W_{(\lambda /2)}\right] }\right] }>0. \end{aligned}$$

$$\square $$

We note that the expected change in distance to the optimum showed in 5.2 is often exponentially small in $\lambda$. Because of this, applying the negative drift theorem is challenging, even when using scaling ([27, Theorem 2]). This is because the negative drift is small and probabilities for large jumps only start to decay at a comparatively large distance.

Nonetheless, here we show that the runtime of the algorithm is infinite in expectation. To show this we rely on the lower additive drift theorem for bounded state spaces (Theorem 2.3) shown by Kötzing and Krejca [29, Theorem 8] which gives a lower bound on the runtime as long as the expected step size is bounded.

### Proof of Theorem 5.1

We aim to use the additive drift theorem with expected bounded step size (Theorem 2.3) to obtain a lower bound $\textrm{E}\mathord {\left[ T\right] }\ge \tau$ that holds for any $\tau \in \mathbb {N}$. Therefore, $\textrm{E}\mathord {\left[ T\right] }=\infty$ otherwise the previous statement would be false for at least some $\tau \in \mathbb {N}$.

By Lemma 5.2$M_t-\textrm{E}_t\mathord {\left[ M_{t+1}\right] }\le \delta$ for any distance $M_t>0$ and any positive $\delta$ meeting the condition *(a)* of Theorem 2.3. For condition *(b)* it is sufficient to note that at any moment the expected step size is bounded by how far the offspring $P_t$ is created from $x_t$ in addition to how far the offspring $Q_t$ is created from $y_t$. Since each offspring is created by adding or subtracting a random variable sampled from a geometric distribution we can bound the expected step size by $2 \textrm{E}\mathord {\left[ W_{(\lambda /2)}\right] }$, where $W_{(\lambda /2)}:=\displaystyle \max _{i\in [\lambda /2]}\{W_i\}$ and $\forall i\in [\lambda /2]$, $W_i\sim \mathcal {G}(p)$. From Lemma 4.1 we know that this is bounded, meeting condition *(b)*. Hence, $\textrm{E}\mathord {\left[ T\right] }\ge M_0 / \delta$ for any $\delta >0$ completing the proof. $\square$

## Fitness Based on Worst Interactions is Efficient

**Fig. 4:**
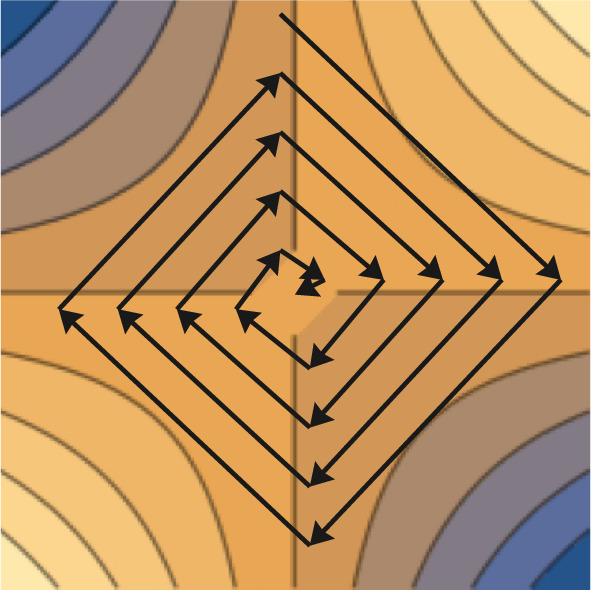
Behaviour of a typical run of ${(1 , \lambda )}~\textrm{CoEA}$ with fitness measure $f^{\textrm{wrs}}$ on Bilinear

In this section we analyse the ${(1 , \lambda )}~\textrm{CoEA}$ using $f^{\textrm{wrs}}$ on Bilinear. The main result of this section is Theorem 6.1, where we show that the algorithm finds a pair of solutions near the optimum efficiently. Even though we do not show that the ${(1 , \lambda )}~\textrm{CoEA}$ using $f^{\textrm{wrs}}$ finds the optimum we believe this already highlights the significant differences in performance between the two fitness measures. The main idea of the proof is that the algorithm moves through each quadrant in a clockwise manner. While inside a quadrant the algorithm maintains its distance to the optimum in expectation and each time it moves from one quadrant to the other the last generation reduces the distance to the optimum. Therefore, after the algorithm traverses through a whole quadrant it has moved towards the optimum in expectation. Figure 4 visualises this behaviour.

### Theorem 6.1

Consider Algorithm 1 with fitness measure $f^{\textrm{wrs}}$ on Bilinear. Let $c>0$ be constant. Define $d:= M_0$, ${{\,\textrm{OPT}\,}}:=\{(x,y)\mid M(x,y)<(8+4 c) \log _{\frac{1}{1-p}}\lambda \}$ and $T:=\min \{t \lambda ^2 \mid P_t\times Q_t\cap {{\,\textrm{OPT}\,}}\ne \emptyset \}$. Let $\lambda =\Theta (d)$, $C\le d^c$. Then, for some constant $\xi$ with probability at least $1-O(1/C)$,

$$\begin{aligned} T \le C \xi \lambda ^2 d^2. \end{aligned}$$

Owing to the large number of different behaviours the fitness measure $f^{\textrm{wrs}}$ have, the analysis of the ${(1 , \lambda )}~\textrm{CoEA}$ using $f^{\textrm{wrs}}$ can quickly become extremely complex. This is particularly true when offspring are created in all four quadrants, as the Manhattan distance to the optimum can increase substantially. To avoid these edge cases, we analyse the first time that the algorithm creates a pair of solutions near the Maximin-solutions (the target set ${{\,\textrm{OPT}\,}}$), making it unlikely for offspring to be created in all four quadrants before the target is reached. From this point, we can obtain a runtime that holds given that the offspring is never in all four quadrants at once, or equivalently they are never created too far from the parents. This constrains the behaviours the fitness measure $f^{\textrm{wrs}}$ have and simplifies the analysis considerably. In the following definition we formalise what we mean by the offspring being far from the parents.

### Definition 6.2

Let $E^{(1)}_t$ denote the event that Algorithm 1 with parents *x*, *y* creates an offspring $x'$ with $\left|x-x'\right|\ge (4+2c)\log _{\frac{1}{1-p}} (\lambda /2)$ or an offspring $y'$ with $\left|y-y'\right|\ge (4+2c)\log _{\frac{1}{1-p}} (\lambda /2)$ at generation *t*.

In addition to simplify further the analysis we divide a run of the algorithm into different intervals that denote when the algorithm moves from one quadrant to the other (not necessarily in a clockwise manner). We call these intervals *blocks*.

### Definition 6.3

(*Blocks*) Let $\tau _{k}:=\min \{t\mid t>\tau _{k-1}\wedge ((x_t-\beta )(x_{\tau _{k-1}}-\beta )<0 \vee (y_t-\alpha )(y_{\tau _{k-1}}-\alpha )<0)\}$ and $\tau _0:=0$. A block comprises all generation in an interval $(\tau _k,\tau _{k+1}]$.

The first step is to show that the algorithm does not spend too much time in one block. This is shown in the following lemma.

### Lemma 6.4

Let $d:=M_{\tau _{k}}$ and ${{\,\textrm{OPT}\,}}:=\{(x,y)\mid M(x,y)<(8+4c) \log _{\frac{1}{1-p}}\lambda \}$. Consider the ${(1 , \lambda )}~\textrm{CoEA}$ using $f^{\textrm{wrs}}$ with $\lambda = O(d)$ starting a block with $d>0$. The probability that the algorithm spends more than $d \log d$ generations before a pair of solutions is created in ${{\,\textrm{OPT}\,}}$, event $E^{(1)}$ happens or a block finishes is at most $d^{-\Omega (\log d)}$.

### Proof

By the definition of a block once the algorithm moves to another quadrant a block finishes. We pessimistically omit all possibilities of moving to another quadrant except by moving to the quadrant that is clockwise to the current one. Additionally we assume that a pair of solutions in ${{\,\textrm{OPT}\,}}$ is never created and event $E^{(1)}$ never happens. Since we look for an upper bound removing stopping conditions only worsens the upper bound. To ease computations we also assume that we are in the second quadrant and by the symmetry of the problem the results apply to all quadrants.

Since the initial Manhattan distance is *d* and we are in the second quadrant, the algorithm starts with a parent $y_{\tau _{k}}$ with $0<y_{\tau _{k}}-\alpha \le d$ and it needs to find a solution *y* with $y-\alpha \le 0$.

To fit the perspective of the additive drift tail bounds [28, Theorem 2.4.7] we use a potential function ${h (y_t) := \min \{0, y_t-\alpha \}}$. Let $\Delta _h:=h (y_t)-h (y_{t+1})$. Given the assumptions during all generations the algorithm will select an offspring $y'$ that satisfies $y'=\min _{y\in Q_t}{y}$, therefore

$$\begin{aligned} \textrm{E}_t\mathord {\left[ y_t- \alpha -(y_{t+1}- \alpha )\right] }=\textrm{E}_t\mathord {\left[ y_t-y_{t+1}\right] } \ge \left( 1/2-e^{-1}\right) \lfloor \log _{\frac{1}{1-p}} (\lambda /2) \rfloor =\Omega (\log \lambda ), \end{aligned}$$

where the inequality follows by Lemma 4.1. We note that $\textrm{E}_t\mathord {\left[ y_t-y_{t+1}\right] } = \textrm{E}_t\mathord {\left[ \Delta _h\right] }$ for all *t* except the last one of the block. For this last step we can bound $h (y_t)\ge 1$ and $h (y_{t+1})=0$. Therefore, $\textrm{E}_t\mathord {\left[ \Delta _h\right] }\ge 1$.

This shows condition (2.24) from [28, Theorem 2.4.7]. We now show condition (2.21) as follows. Let $W_{(\lambda /2)}:=\displaystyle \max _{i\in [\lambda /2]}\{W_i\}$ with $W_i\sim \mathcal {G}(p)$, then

$$\begin{aligned} \textrm{Pr}\left[ \left|\Delta _h\right|>j\right] \le \textrm{Pr}\left[ y_t-y_{t+1}\ge j\right] = \textrm{Pr}\left[ W_{(\lambda /2)}\ge j\right] . \end{aligned}$$

By Lemma 4.3 (10) with $\delta = 2 \left( \frac{1}{1-p}\right) ^j/\lambda$ we obtain,

$$\begin{aligned} \textrm{Pr}\left[ \left|\Delta _h\right|>j\right] \le \frac{\lambda }{2\left( \frac{1}{1-p}\right) ^j}, \end{aligned}$$

and the last condition (2.21) from [28, Theorem 2.4.7] is met with $\eta =\frac{p}{1-p}$ and $r=\lambda /2$.

Let, $\tau$ be the time the algorithm spends in a block, then the additive drift tail bounds yield,

$$\begin{aligned}&\textrm{Pr}\left[ \tau \ge d \log d\right] \\&\quad \le \exp {\left( \left( -\frac{\left( \frac{p}{1-p}\right) (d \log d-d))}{8} \right) \left( \frac{\left( \frac{p}{1-p}\right) ^2(d \log d-d)}{16 \lambda d}\right) \right) }\\&\quad =\exp {\left( -\Omega (\log ^2 d)\right) }=d^{-\Omega (\log d)}. \end{aligned}$$

$$\square $$

Now we show a tail bound for number of evaluations made before a pair of points in ${{\,\textrm{OPT}\,}}$ is found or until the event *E*(1) happens.

### Lemma 6.5

Consider Algorithm 1 with fitness measure $f^{\textrm{wrs}}$ on Bilinear. Let $c>0$ be constant. Define $d:= M_0$, ${{\,\textrm{OPT}\,}}:=\{(x,y)\mid M(x,y)<(8+4c) \log _{\frac{1}{1-p}}\lambda \}$ and $\overline{T}:=\min \{t\lambda ^2 \mid (P_t\times Q_t\cap {{\,\textrm{OPT}\,}}\ne \emptyset )\vee E^{(1)}\}$. Let $\lambda = \Theta (d)$, $C\le d^c$. Then, for some constant $\xi$ with probability at least $1-2/C$, $\overline{T}\le C \xi \lambda ^2 d^2$.

### Proof

Given that we want an upper bound for $\overline{T}$ we pessimistically assume the event $E^{(1)}_t$ does not happen for all $t<\overline{T}$. For brevity we write $\Delta _x:=x_t-x_{\min }=x_{\max }-x_t$ and $\Delta _y:=y_t-y_{\min }=y_{\max }-y_t$ and note that $\textrm{E}\mathord {\left[ \Delta _x\right] }=\textrm{E}\mathord {\left[ \Delta _y\right] }=\textrm{E}\mathord {\left[ W_{(\lambda /2)}\mid \overline{E^{(1)}_t}\right] }$.

Now we divide the runtime in blocks as defined in Definition 6.3 and aim to use the additive drift theorem that allows overshooting the target (Theorem 2.2 [29, Theorem 7]) to show that it takes the algorithm at most *d* blocks in expectation to reach the stopping condition. We use the shifted Manhattan distance $G_t:=g(x_t,y_t)=M(x_t,y_t)-\log _{\frac{1}{1-p}}d$ as a potential function, therefore $G_t\ge -\log _{\frac{1}{1-p}}d$ and the first condition of the additive drift theorem (Theorem 2.2) is met.

**Fig. 5:**
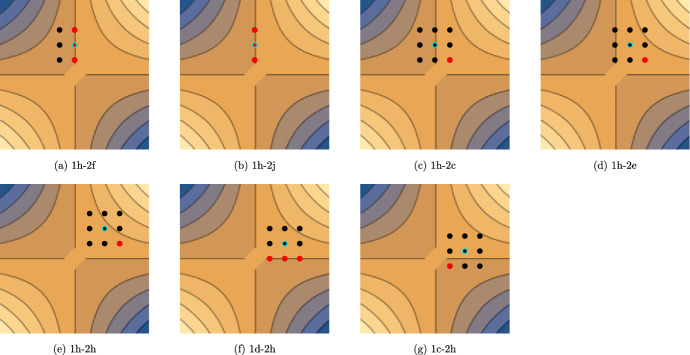
Possible configurations of the offspring populations $P_t\times Q_t$ with the parents starting in the second quadrant. The parent is shown as a hollow cyan circle, the offspring with highest fitness with respect to $f^{\textrm{avg}}$ is coloured in red and all the other offspring are coloured black

Now we proceed to show the second condition. As in previous sections we assume that the algorithm is in the second quadrant and note that by the symmetry of the problem the results apply to all quadrants. This, combined with the assumptions on $\overline{E^{(1)}_t}$ imply that at any generation $t<\overline{T}$ the offspring populations $P_t\times Q_t$ follow the cases 1 h–2f, 1 h–2j, 1 h–2c, 1 h–2e, 1 h-2 h, 1d–2 h, 1c–2 h, from Lemma 3.2. These cases are illustrated in Fig. 5.

For the first two cases (1 h–2f, 1 h–2j), the selected offspring $x'$ is a copy of the parent *x*, and the offspring $y'$ is selected at random from all offspring in $Q_t$ then there is the same probability of having a value $y_t+i$ and $y_t-i$ for all $i\in \mathbb {N}$ then in expectation $y_t=y_{t+1}$ and $\textrm{E}\mathord {\left[ y_t-y_{t+1}\right] }=0$. Therefore, for these cases the expected change in distance to the optimum is 0. We note that these cases can only happen in the first generations of a block and once any other case happens they cannot occur anymore.

All the next generations, up until $y_t\le \alpha + (8+4c) \log _{\frac{1}{1-p}}\lambda$ will satisfy the conditions of cases 1 h–2c, 1 h–2e or 1 h–2 h because of the assumption that $E^{(1)}_t$ never happens. By Lemma 3.2 in these generations the algorithm will always select $x_{t+1}=x_{\max }=x_{t}+\Delta _x$ and $y_{t+1}=y_{\min }=y_{t}-\Delta _y$. Recall that $\textrm{E}\mathord {\left[ \Delta _x\right] }=\textrm{E}\mathord {\left[ \Delta _y\right] }$ therefore the expected change in distance to the optimum in these generations is 0.

Let $t^*$ be the first generation with $y_{t^*}\le \alpha + (8+4c) \log _{\frac{1}{1-p}}\lambda$, $d^*:=y_{t^*} - \alpha$ and $T^*=\inf \{t\mid y_t \le 0\}$. As before, in all these generations but the last one the offspring meet the cases 1 h–2c, 1 h–2e or 1 h–2 h and the offspring of the last generation meet either the cases 1c–2 hor 1d–2 h.

We will divide the proof in two cases, when the last generation meets 1c–2 h and when it meets 1d–2 h.

To analyse the case 1c–2 h we first need to show that $(\textrm{E}\mathord {\left[ T^*\right] }-1)\textrm{E}\mathord {\left[ \Delta _y\right] }\le d^*$. To achieve this, we compute a bound for $\textrm{E}\mathord {\left[ T^*\right] }$ using the additive drift theorem that allows overshooting the target (Theorem 2.2) with a potential function $F_t:=y_t-\alpha$. To show the first condition of Theorem 2.2 it is sufficient to note that we are conditioning on $\overline{E^{(1)}_t}$, therefore for all $t\le T^*$, $F_t \ge -(8+4c) \log _{\frac{1}{1-p}}\lambda$. For the second condition we note that by Lemma 3.2 in all these generations the drift of $y_t$ towards $\alpha$ is $\Delta _y$. Then, by the additive drift theorem $\textrm{E}\mathord {\left[ T^*\right] }\le \frac{d^*-\textrm{E}\mathord {\left[ F_{T^*}\mid F_0\right] }}{\textrm{E}\mathord {\left[ \Delta _y\right] }}$. Now we compute the expected overshoot $\textrm{E}\mathord {\left[ F_{T^*}\mid F_0\right] }$ using Lemmas 4.1 and 4.2.

$$\begin{aligned} \textrm{E}\mathord {\left[ F_{T^*}\mid F_0\right] }&=k-\textrm{E}\mathord {\left[ \Delta _y\mid \Delta _y>k\right] } \ge - \textrm{E}\mathord {\left[ \Delta _y\right] } + \Omega (\log \lambda ), \end{aligned}$$

where *k* is $y_{T^*-1}-\alpha$. By Lemma 4.1$\textrm{E}\mathord {\left[ \Delta _y\right] }=\Theta (\log \lambda )$, then this shows that for some constant $0<B<1$, $\textrm{E}\mathord {\left[ T^*\right] }\le \frac{d^*}{\textrm{E}\mathord {\left[ \Delta _y\right] }}+ B \le \frac{d^*}{\textrm{E}\mathord {\left[ \Delta _y\right] }}+1$ and rearranging we obtain

20 $$\begin{aligned} (\textrm{E}\mathord {\left[ T^*\right] }-1)\textrm{E}\mathord {\left[ \Delta _y\right] }\le d^*. \end{aligned}$$

Now, we return to analyse the case 1c–2 h. The expected change in distance from *y* during the $T^*$ generations is at least $d^*$ minus the overshoot in the last generation. The expected change in distance from *x* is $-(\textrm{E}\mathord {\left[ T^*\right] }-2)\textrm{E}\mathord {\left[ \Delta _x\right] }$ because all generations but the last move away from the optimum by $\textrm{E}\mathord {\left[ \Delta _x\right] }$ and the last moves towards the optimum by $\textrm{E}\mathord {\left[ \Delta _x\right] }$. From Eq. (20) we know that $(\textrm{E}\mathord {\left[ T^*\right] }-1)\textrm{E}\mathord {\left[ \Delta _x\right] }\le d^*$ then $-(\textrm{E}\mathord {\left[ T^*\right] }-2)\textrm{E}\mathord {\left[ \Delta _x\right] }\ge -d^* + \textrm{E}\mathord {\left[ \Delta _x\right] }$. Since *p* is constant, by Lemmas 4.1 and 4.2 the expected overshoot is smaller than $\textrm{E}\mathord {\left[ \Delta _x\right] }$ by $\Omega (\log \lambda )$. Therefore, the expected change in distance during the block for this case is at least

$$\begin{aligned} d^*- \textrm{E}\mathord {\left[ \text {overshoot}\right] } -d^* + \textrm{E}\mathord {\left[ \Delta _x\right] } =\Omega (\log \lambda ). \end{aligned}$$

For the case 1d–2 h, the expected change in distance from *y* is exactly $d^*$. The expected change in distance from *x* is $-(\textrm{E}\mathord {\left[ T^*\right] }-1)\textrm{E}\mathord {\left[ \Delta _x\right] }$ because all generations but the last move away from the optimum and the last one selects an offspring that in expectation is at the same distance than its parent because the probability of creating an offspring $x'$ from the parent *x* with $x'-x=c$ is the same as $x'-x=-c$ for all *c*. As before by the additive drift theorem $\textrm{E}\mathord {\left[ T^*\right] }\le d^*/\textrm{E}\mathord {\left[ \Delta _y\right] }+B$ and $(\textrm{E}\mathord {\left[ T^*\right] }-1)\textrm{E}\mathord {\left[ \Delta _x\right] }\le d^*-\textrm{E}\mathord {\left[ \Delta _x\right] } (1-B)$. Therefore, the expected change in distance during the block for this case is at least

$$\begin{aligned} d^*-d^*+\textrm{E}\mathord {\left[ \Delta _x\right] } (1-B)=\Theta (\log \lambda ). \end{aligned}$$

This shows that the second condition of Theorem 2.2 holds for $\delta = \Theta (\log \lambda )= \xi ' \log d$ for some constant $\xi '$. Therefore, by the additive drift theorem (Theorem 2.2), for $\xi =1/\xi '=O(1)$ the algorithm needs at least $\xi d/ \log d$ blocks in expectation to find a pair of solutions $(x,y)\in {{\,\textrm{OPT}\,}}$. By Markov’s inequality $\textrm{Pr}\left[ \overline{T}\ge C \xi d/\log d\right] \le 1/C$ or equivalently, $\textrm{Pr}\left[ \overline{T}< C \xi d /\log d\right] \ge 1-1/C$.

By Lemma 6.4 each block takes at most $d \log d$ generations during a block with probability at least $1-d^{-\Omega (\log d)}$. By a union bound over all blocks the probability that any block takes at most $d \log d$ is at least $1-(C d/\log d)\cdot d^{-\Omega (\log d)}$. Therefore, the stopping condition is met within $C \xi d^2$ generations with probability

$$\begin{aligned} \left( 1-\frac{1}{C}\right) \left( 1-\frac{C \cdot d}{d^{\Omega (\log d)}\log d}\right) \ge 1-\frac{1}{C}-\frac{C \cdot d}{d^{\Omega (\log d)} \log d} \ge 1-\frac{2}{C}, \end{aligned}$$

Where the last inequality holds because $C\le d^c$ for some constant *c*. Finally, each generation uses $\lambda ^2$ evaluations. $\square$

We now show that event $E^{(1)}$ is sufficiently rare for it to not happen with high probability during a run of the algorithm.

### Lemma 6.6

Let $\lambda =\Omega (d)$, $d:=M_0$, $C\le d^c$ for a $c>0$. The probability that after $C \cdot d^2$ generations from Algorithm 1 using the geometric mutation operator at least one generation starts with parents *x*, *y* and creates an offspring $x'$ with $\left|x-x'\right|\ge (4+2c)\log _{\frac{1}{1-p}} (\lambda /2)$ or an offspring $y'$ with $\left|y-y'\right|\ge (4+2c)\log _{\frac{1}{1-p}} (\lambda /2)$ is at most $O\left( d^{-c}\right)$

### Proof

By Lemma 4.3 (10) with $\psi =\lambda /2$, the probability of creating an offspring $x'$ with distance $\left|x-x'\right|\ge (4+2c)\log _{\frac{1}{1-p}} (\lambda /2)$ is $(2/\lambda )^{3+2c}$. The same applies for $y'$. Since each event has two trials for $x'$ and two for $y'$ the sought probability for only one generation is $4 (2/\lambda )^{3+2c}$.

By a union bound after $C \cdot d^2$ generations the probability of the event happening is

$$\begin{aligned} O\left( \frac{2^{5+2c} d^{2+c}}{\lambda ^{3+2c}}\right) = O\left( d^{-c}\right) . \end{aligned}$$

$$\square $$

With the previous lemmas we are now able to prove Theorem 6.1.

### Proof of Theorem 6.1

By Lemma 6.5 with probability at least $1-2/C$, the algorithm will find a pair of points in ${{\,\textrm{OPT}\,}}$ or event $E^{(1)}$ will happen in $C \xi d^2$ generations. By Lemma 6.6 with probability $1-O(d^{-c})$ event $E^{(1)}$ will not happen in $C \xi d^2$ generations therefore, with probability at least

$$\begin{aligned} \left( 1-\frac{2}{C}\right) (1-O(d^{-c}))\ge \left( 1-\frac{2}{C}\right) \left( 1-O\left( \frac{1}{C}\right) \right) = 1-O\left( \frac{1}{C}\right) , \end{aligned}$$

the algorithm will find a pair of points in ${{\,\textrm{OPT}\,}}$ in $C \xi d^2$ generations and $C \xi \lambda ^2 d^2$ evaluations. $\square$

## Experiments

Although our theoretical results have given us important insights of how the fitness measures affect the performance of CoEAs, they are limited to only one search space ($\mathcal {X} =\mathcal {Y} =\mathbb {Z}$). It is unclear if our results translate to other search spaces. In this section, we conduct an experimental analysis that aims to complement our theoretical results with precise runtime results for the ${(1 , \lambda )}~\textrm{CoEA}$ with different fitness measures on Bilinear problems defined over three different search spaces ($\mathcal {X} =\mathcal {Y} =\mathbb {Z}$, $\mathcal {X} =\mathcal {Y} =\mathbb {R}$ and $\mathcal {X} =\mathcal {Y} =\{0,1\}^n$). For the search space $\mathcal {X} =\mathcal {Y} =\{0,1\}^n$ we use the definition of Bilinear used by [16] with $n=500$ in all experiments and two parameter values ($\alpha =\beta =0.5$ and $\alpha =0.8, \beta =0.75$). We note that the mutation operator in the case $\alpha =\beta =0.5$ has an inherent *genetic drift* towards the optimum (*n*/2). Therefore the case $\alpha =\beta =0.5$ should be easy to optimise even for a ${(1 , \lambda )}~\textrm{CoEA}$ that selects a u. a. r. offspring every time.

For the three different search spaces the ${(1 , \lambda )}~\textrm{CoEA}$ uses the following mutation operators:

- $\mathcal {X} =\mathcal {Y} =\mathbb {Z}$ - Geometric mutation (random direction and $p=1/2$): For a parent *x*, the offspring $x'=x+\mathcal {G} (1/2)$ with probability 1/2 and $x'=x-\mathcal {G} (1/2)$ otherwise.
- $\mathcal {X} =\mathcal {Y} =\mathbb {R}$ - Gaussian mutation (step size of 1): For a parent *x*, the offspring $x'=x+\mathcal {N}(0,1)$.
- $\mathcal {X} =\mathcal {Y} =\{0,1\}^n$ - Standard bit mutation ($p=1/n$): An offspring is created by copying the parent and flipping each bit with probability 1/*n*.

All experiments comprise of 100 runs for each algorithm-problem pair, recording the number of evaluations (interactions) to reach the optimum. Each run is limited to $10^{9}$ evaluations, if the run does not find the optimum within this time limit, $10^{9}$ is reported as the runtime. For the search space $\mathcal {X} =\mathcal {Y} =\mathbb {R}$ we assume the optimum is found if both $x\in [\beta -0.1, \beta +0.1]$ and $y\in [\alpha -0.1, \alpha +0.1]$. The figures shown in this section are boxplots where the box represents the quartiles (25th to 75th percentiles) and the whiskers denote the rest of the distribution, excluding outliers, which are shown as individual points.

**Fig. 6:**
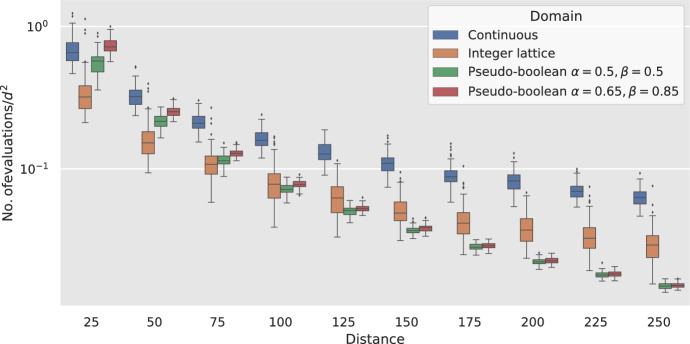
Runtime of the ${(1 , \lambda )}~\textrm{CoEA}$ with fitness measure $f^{\textrm{wrs}}$ on Bilinear

We first explore the fitness measure $f^{\textrm{wrs}}$. In Fig. 6 we show in the *x*-axis the initial Manhattan distance to the optimum^4^ and in the *y*-axis the runtime divided by $d^2$ and log-scaled. Given that the number of evaluations are normalised by $d^2$ we can appreciate that for the distances studied and all search spaces, the runtime seem to grow slower than $d^2$. This indicate that our bounds might not be tight or that they are tight but only for large initial distances *d*. Nonetheless, these experiments are evidence that our theoretical insights on $f^{\textrm{wrs}}$ do translate to other search spaces. Additionally, for the pseudo-Boolean search domains, the runtimes tend to be faster, which could be attributed to the inherent genetic drift aiding the search process.

**Fig. 7:**
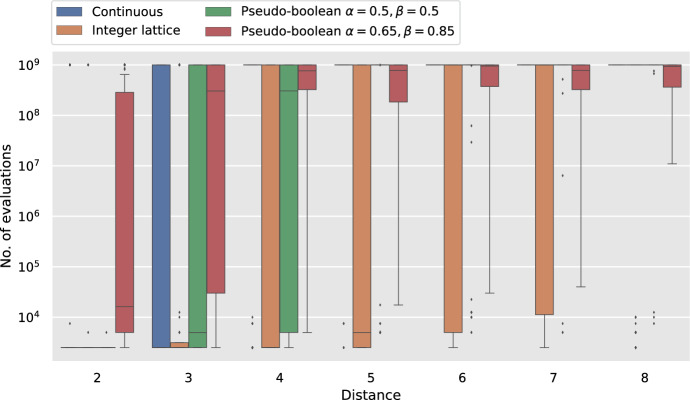
Runtime of the ${(1 , \lambda )}~\textrm{CoEA}$ with fitness measure $f^{\textrm{avg}}$ on Bilinear

Similarly, the experimental results for the fitness measure $f^{\textrm{avg}}$ (Fig. 7) show that our theoretical results do translate to other search spaces. For the continuous and integer lattice domains, there is always at least one run that does not find the optimum. This is most likely because the algorithm did not generate the optimum when the current pair of solutions was close to it, and due to the drift, it subsequently moved further and further away. For these experiments it is particularly interesting to see that the runtime *explodes* even for small initial distances to the optimum for all search domains.

## Conclusions

We have shown how the selection of fitness measure dramatically affects the performance of the ${(1 , \lambda )}~\textrm{CoEA}$ on Bilinear. Both fitness measures studied here present a cyclic behaviour on this problem. Using the average payoff of interactions worsens this behaviour resulting in an expected infinite time to find an optimal solution. On the other hand using the worst payoff alleviates the problem resulting in an efficient optimisation.

An important insight from our theoretical analysis is that both fitness measures tend to maintain the distance to the optimum in most generations and what differentiates them is the behaviour during the generation where the current solutions are part of more than one quadrant of the search space. While using the worst interaction as fitness decreases the distance to the optimum averaging interactions increases it. We hope that this insight inspires the design of better CoEAs that exploit this behaviour.

Although our theoretical results only directly apply for the studied class of functions. Vlatakis-Gkaragkounis et al. [22] argues that the structure of the Bilinear problem class is similar to the underlying structure of many applications such as the training of GANs. We believe that the techniques used and developed can be useful to analyse these similar problems and more importantly the insights obtained by our analysis are helpful to approach these problems too.

Nonetheless, it remains an open problem, whether the results shown here apply to intransitive problems with a different underlying structure and how do other fitness measures affect the performance of CoEAs on intransitive problems. Furthermore, an interesting venue for future work is to theoretically analyse other classes of problems to examine whether CoEAs are affected by the fitness measure on these problems to the same extent as seen in this work.

## Footnotes

1 Studied in Cooperative CoEAs.

2 Other than small tailored examples showing fitness measures can be misleading [13].

3 Since CoEAs are comparison based, the average or the sum of the evaluations result in the same behaviour.

4 For the search space $\mathcal {X} =\mathcal {Y} =\{0,1\}^n$ the Manhattan distance is defined as the sum of the Hamming distances of both *x* and *y* to an optimal solution.
